# Impact of preprocedural coronary flow grade on duration of dual antiplatelet therapy in acute myocardial infarction

**DOI:** 10.1038/s41598-021-91130-5

**Published:** 2021-06-03

**Authors:** Yong Hoon Kim, Ae-Young Her, Byeong-Keuk Kim, Sung-Jin Hong, Chul-Min Ahn, Jung-Sun Kim, Young-Guk Ko, Donghoon Choi, Myeong-Ki Hong, Yangsoo Jang

**Affiliations:** 1 Division of Cardiology, Department of Internal Medicine, Kangwon National University School of Medicine, Chuncheon, Republic of Korea; 2 Division of Cardiology, Severance Cardiovascular Hospital, Yonsei University College of Medicine, Seoul, Republic of Korea

**Keywords:** Cardiology, Medical research

## Abstract

We investigated the impact of pre-percutaneous coronary intervention (pre-PCI) thrombolysis in myocardial infarction (TIMI) flow grade (pre-TIMI) on 3-month (3-mo) and 12-mo of dual antiplatelet therapy (DAPT) in patients with acute myocardial infarction (AMI). This was a post hoc analysis of the TICO trial. A total of 2083 patients with AMI (pre-TIMI 0/1: n = 1143; pre-TIMI 2/3: n = 940) were evaluated. The primary outcome was the occurrence of net adverse clinical events (NACE), defined as a composite of TIMI major bleeding and major adverse cardiac and cerebrovascular events (MACCE) within 12-mo following PCI. The secondary outcomes were the occurrence of the individual components of TIMI bleedings and MACCE. In the pre-TIMI 0/1 group, the primary and second outcomes were not significantly different between the 3-mo and 12-mo DAPT groups. However, in the pre-TIMI 2/3 group, the occurrences of TIMI minor (adjusted hazard ratio [aHR]: 0.294; *p* = 0.016) and major or minor bleeding (aHR: 0.483; *p* = 0.014) on intention-to-treat analysis were significantly higher in the 12-mo than in the 3-mo DAPT group. The occurrence of MACCE was similar between the two groups. A higher bleeding tendency in 12-mo DAPT compared with 3-mo DAPT was more obvious in the pre-TIMI 2/3 group than in the pre-TIMI 0/1 group.

Clinical Trial Registration: URL: http://www.clinicaltrials.gov. Unique identifier: NCT02494895.

## Introduction

Current guidelines^1,2^ recommend 12-month (12-mo) dual antiplatelet therapy (DAPT) consisting of aspirin with a P2Y_12_ inhibitor after percutaneous coronary intervention (PCI) in patients with acute myocardial infarction (AMI). However, more recent reports^3,4^ have shown that 3-month (3-mo) duration of DAPT could reduce hemorrhagic risk without increasing the risk of ischemic events. Platelets play a key role in the development of acute coronary syndrome^5^, and platelet activation and plugging are of significant importance in the development of impaired pre-PCI flow because platelet-mediated release of vasoactive mediators increases platelet-rich thrombi formation^5,6^. Under the circumstance where blood supply is completely absent, available oxygen in the ischemic zone of the myocardium disappears within seconds. Hence, after a certain duration of complete ischemia, there is no treatment modality that can salvage ischemic myocardium^7^. However, cardiomyocytes that are exposed to low residual oxygen levels may be able to maintain sufficient adenosine triphosphate to survive for an extended period, even if the amount of adenosine triphosphate is insufficient to allow their contraction^7^. Hence, we might think that patients with pre-PCI thrombolysis in myocardial infarction (pre-PCI TIMI) flow grade 0/1 (pre-TIMI 0/1) or pre-TIMI 2/3^8^ are in a meaningful different situation. Compared to patients with pre-TIMI 0/1, those with pre-TIMI 2/3 have a lower incidence of cardiogenic shock and improved early and late left ventricular ejection fraction (LVEF) through preservation of flow to the infarct zone, with consequent preservation of myocardial viability^9,10^. However, comparative clinical outcomes between short-term and standard 12-mo DAPT according to pre-TIMI in patients with AMI has not been reported. Therefore, the authors thought that the reevaluation of safety and efficacy of 3-mo and 12-mo DAPT according to the different pre-TIMI in patients with AMI could provide beneficial information to treat those patients. In this post hoc analysis of the TICO trial (Ticagrelor Monotherapy After 3 Months in the Patients Treated With New Generation Sirolimus-eluting Stent for Acute Coronary Syndrome)^4^, we compared 1-year clinical outcomes between these 2 different antiplatelet strategies in patients with AMI, after new-generation drug-eluting stent (DES) implantation.

## Results

### Baseline characteristics

Detailed information on antiplatelet therapy during the study period, causes of non-adherence to the allocated treatment, and medications during the study period are shown in Supplementary materials 1, 2, and 3. Table 1 shows the baseline characteristics of the study population. In both patients with pre-TIMI 0/1 and 2/3, the mean age, the number of males, and the mean value of LVEF were similar between the 3-mo and 12-mo DAPT groups. In patients with pre-PCI TIMI 0/1, the number of patients with a history of prior MI, the mean value of estimated glomerular filtration rate (eGFR), and the prescription rate of angiotensin receptor blockers (ARB) and calcium channel blockers (CCB) were significantly higher in the 3-mo DAPT group than in the 12-mo DAPT group. In contrast, the mean value of serum creatinine, and the prescription rates of beta-blockers and angiotensin converting enzyme inhibitors (ACEI) were higher in the 12-mo DAPT group. In patients with pre-TIMI 2/3, eGFR was higher in the 3-mo DAPT group, and the prescription rate of ticagrelor and beta-blockers was higher in the 12-mo DAPT group. Supplementary material 4 shows the baseline characteristics according to 3-mo or 12-mo DAPT strategies. Supplementary materials 5 and 6 show the baseline characteristics of the as-treated population.

**Table 1:** Baseline clinical, laboratory, angiographic and procedural characteristics.

Variables	Pre-PCI TIMI 0/1 (n = 1143)				Pre-PCI TIMI 2/3 (n = 940)			
	Total	Ticagrelor monotherapy after 3-mo DAPT (n = 582)	Ticagrelor-based 12-mo DAPT group (n = 561)	*p*	Total	Ticagrelor monotherapy after 3-mo DAPT (n = 475)	Ticagrelor-based 12-mo DAPT group (n = 465)	*p*
Age (years)	58.7 ± 10.8	58.5 ± 10.8	58.8 ± 10.7	0.633	61.5 ± 11.0	61.3 ± 10.9	61.7 ± 11.1	0.574
Male, n (%)	957 (83.7)	476 (81.8)	481 (85.7)	0.070	760 (80.9)	386 (81.3)	374 (80.4)	0.746
LVEF (%)	49.4 ± 10.7	49.9 ± 10.9	48.9 ± 10.5	0.166	54.6 ± 11.4	54.8 ± 11.0	54.4 ± 11.8	0.666
BMI (kg/m^2^)	24.9 ± 3.2	24.9 ± 3.2	25.0 ± 3.2	0.687	24.8 ± 3.3	24.7 ± 3.2	24.8 ± 3.4	0.638
Hypertension, n (%)	504 (44.1)	261 (44.8)	243 (43.3)	0.603	490 (52.1)	246 (51.8)	244 (52.5)	0.834
Diabetes mellitus, n (%)	247 (21.6)	130 (22.3)	117 (20.9)	0.543	278 (29.6)	136 (28.6)	142 (30.5)	0.568
Dyslipidemia, n (%)	652 (57.0)	332 (57.0)	320 (57.0)	0.999	566 (60.2)	288 (60.6)	278 (59.8)	0.791
Prior MI, n (%)	32 (2.8)	23 (4.0)	9 (1.6)	0.026	36 (3.8)	20 (4.2)	16 (3.4)	0.612
Prior PCI, n (%)	65 (5.7)	40 (6.9)	25 (4.5)	0.096	74 (7.9)	35 (7.4)	39 (8.4)	0.628
Prior CABG, n (%)	5 (0.4)	3 (0.5)	2 (0.4)	0.684	6 (0.6)	1 (0.2)	5 (1.1)	0.120
Prior HF, n (%)	19 (1.7)	7 (1.2)	12 (2.1)	0.252	12 (1.3)	8 (1.7)	4 (0.9)	0.385
Prior stroke, n (%)	42 (3.7)	18 (3.1)	24 (4.3)	0.346	41 (4.4)	17 (3.6)	24 (5.2)	0.265
Current smokers, n (%)	523 (45.8)	254 (43.6)	269 (48.0)	0.154	372 (39.6)	188 (39.6)	184 (39.6)	0.980
White blood cell (× 10^9^/L)	10.9 ± 4.0	10.8 ± 4.3	10.9 ± 3.6	0.811	9.3 ± 3.4	9.2 ± 3.3	9.5 ± 3.4	0.107
Hemoglobin (g/dL)	14.6 ± 1.7	14.5 ± 1.7	14.7 ± 1.7	0.185	14.2 ± 1.8	14.2 ± 1.8	14.2 ± 1.8	0.937
Platelet (× 10^9^/L)	245.1 ± 62.6	244.8 ± 60.0	245.4 ± 65.2	0.865	241.7 ± 71.4	237.5 ± 65.6	246.0 ± 76.7	0.067
Peak CK-MB (mg/dL)	476.0 ± 950.8	476.7 ± 932.7	475.3 ± 970.1	0.980	267.2 ± 718.9	311.3 ± 920.4	222.2 ± 518.9	0.330
Peak troponin-I (ng/mL)	32.8 ± 27.9	38.5 ± 32.0	27.0 ± 23.0	0.482	17.2 ± 36.0	16.3 ± 27.4	18.1 ± 43.0	0.415
Serum creatinine (mg/L)	1.01 ± 0.76	0.97 ± 0.55	1.06 ± 0.93	0.043	1.07 ± 0.99	1.02 ± 0.84	1.12 ± 1.11	0.097
eGFR (mL/min/1.73m^2^)	77.0 ± 22.4	78.5 ± 22.6	75.4 ± 22.1	0.017	76.7 ± 27.1	78.4 ± 29.1	74.9 ± 24.9	0.048
**Clinical presentation**								
NSTEMI	360 (31.5)	190 (32.6)	170 (30.3)	0.394	648 (68.9)	338 (71.2)	310 (66.7)	0.137
STEMI	783 (68.5)	392 (67.4)	391 (69.7)	0.394	292 (31.1)	137 (28.8)	155 (33.3)	0.137
**Antithrombotic drug before PCI**								
Unfractionated heparin, n (%)	810 (70.9)	406 (69.8)	404 (72.0)	0.402	652 (69.4)	334 (70.3)	318 (68.4)	0.521
LMWH, n (%)	95 (8.3)	47 (8.1)	48 (8.6)	0.830	86 (9.1)	44 (9.3)	42 (9.0)	0.910
Glycoprotein IIb/IIIa inhibitors	147 (12.9)	73 (12.5)	74 (13.2)	0.744	29 (3.1)	16 (3.4)	13 (2.8)	0.707
**Antiplatelet drug before PCI**								
Aspirin, n (%)	1099 (96.2)	561 (96.4)	538 (95.9)	0.666	912 (97.0)	463 (97.5)	449 (96.6)	0.410
Clopidogrel, n (%)	219 (19.2)	123 (21.1)	96 (17.1)	0.098	328 (34.9)	175 (36.8)	153 (32.9)	0.218
Ticagrelor, n (%)	951 (83.2)	480 (82.5)	471 (84.0)	0.503	693 (73.7)	335 (70.5)	358 (77.0)	0.024
Prasugrel, n (%)	5 (0.4)	2 (0.3)	3 (0.5)	0.681	1 (0.1)	1 (0.2)	0	0.322
**Other discharge medications**								
Beta-blockers, n (%)	821 (71.8)	389 (66.8)	432 (77.0)	< 0.001	638 (67.9)	304 (64.0)	334 (71.8)	0.010
ACE inhibitors, n (%)	603 (46.0)	253 (43.5)	295 (52.6)	0.002	391 (41.6)	188 (39.6)	203 (43.7)	0.209
ARBs, n (%)	230 (20.1)	138 (23.7)	92 (16.4)	0.002	248 (26.4)	125 (26.3)	123 (26.5)	0.962
CCBs, n (%)	97 (8.5)	66 (11.3)	31 (5.5)	< 0.001	119 (12.7)	59 (12.4)	60 (12.9)	0.845
Statin, n (%)	1124 (98.3)	572 (98.3)	552 (98.4)	0.880	921 (98.0)	463 (97.5)	458 (98.5)	0.266
*Angiographic and procedural characteristics*								
**Infarct-related artery**								
LM, n (%)	9 (0.8)	6 (1.0)	3 (0.5)	0.507	33 (3.5)	18 (3.8)	15 (3.2)	0.724
LAD, n (%)	535 (46.8)	273 (46.9)	262 (46.7)	0.953	483 (51.4)	247 (52.0)	236 (50.8)	0.702
LCx, n (%)	204 (17.8)	94 (16.2)	110 (19.6)	0.142	154 (16.4)	87 (18.3)	67 (14.4)	0.113
RCA, n (%)	395 (34.6)	209 (35.9)	186 (32.2)	0.351	270 (28.7)	123 (25.9)	147 (31.6)	0.061
Primary PCI, n (%)	666 (58.3)	333 (57.2)	333 (59.4)	0.463	300 (31.9)	145 (30.5)	155 (33.3)	0.364
Bifurcation lesion, n (%)	124 (10.8)	54 (9.3)	70 (12.5)	0.087	180 (19.1)	86 (18.1)	94 (20.2)	0.456
**Extent of CAD**								
Single-vessel disease, n (%)	544 (47.6)	276 (47.4)	268 (47.8)	0.906	394 (41.9)	198 (41.7)	196 (42.2)	0.885
Two-vessel disease, n (%)	353 (30.9)	187 (32.1)	166 (29.6)	0.370	312 (33.2)	163 (34.3)	149 (32.0)	0.489
≥ Three-vessel, n (%)	246 (21.5)	119 (20.4)	127 (22.6)	0.388	234 (24.9)	114 (24.0)	120 (25.8)	0.547
Transfermoral approach, n (%)	663 (58.0)	343 (58.9)	320 (57.0)	0.517	418 (44.5)	203 (42.7)	215 (46.2)	0.294
Treated lesions per patient	1.19 ± 0.45	1.20 ± 0.46	1.19 ± 0.45	0.696	1.25 ± 0.51	1.27 ± 0.54	1.24 ± 0.49	0.505
Multi-lesion intervention, n (%)	191 (16.7)	100 (17.2)	91 (16.2)	0.692	208 (22.1)	107 (22.5)	101 (21.7)	0.814
Multi-vessel intervention, n (%)	148 (12.9)	77 (13.2)	71 (12.7)	0.792	176 (18.7)	89 (18.7)	87 (18.7)	0.991
Total number of stents per patient	1.33 ± 0.63	1.33 ± 0.64	1.33 ± 0.62	0.967	1.35 ± 0.66	1.38 ± 0.71	1.32 ± 0.61	0.191
Stent diameter, mean (mm)	3.16 ± 0.43	3.18 ± 0.43	3.14 ± 0.42	0.132	3.18 ± 0.45	3.15 ± 0.46	3.21 ± 0.45	0.034
Total stent length per patient (mm)	35.1 ± 19.4	34.8 ± 19.6	36.4 ± 19.1	0.617	32.7 ± 20.0	33.1 ± 20.6	32.3 ± 19.4	0.549
PRECISE-DAPT score	21.6 ± 19.5	21.3 ± 20.1	21.8 ± 19.0	0.671	21.9 ± 19.9	21.8 ± 20.8	22.0 ± 19.0	0.823
≥ 25, n (%)	244 (21.3)	124 (21.3)	120 (21.4)	0.972	232 (24.7)	122 (25.7)	110 (23.7)	0.471

Values are mean ± SD or n (%). The *p* values for continuous data obtained from analysis of the unpaired t-test. The *p* values for categorical data obtained from chi-square test. *Pre-PCI* pre-percutaneous coronary intervention, *TIMI* Thrombolysis In Myocardial Infarction, *DAPT* dual antiplatelet therapy, *LVEF* left ventricular ejection fraction, *BMI* body mass index, *MI* myocardial infarction, *PCI* percutaneous coronary intervention, *CABG* coronary artery bypass graft, *HF* heart failure, *CK-MB* creatine kinase myocardial band, *eGFR* estimated glomerular filtration rate, *NSTEMI* non-ST-elevation MI, *LMWH* low-molecular weight heparin, *ACE* angiotensin converting enzyme, *ARB* angiotensin receptor blocker, *CCB* calcium channel blocker, *LM* left main coronary artery, *LAD* left anterior descending coronary artery, *LCx* left circumflex coronary artery, *RCA* right coronary artery, *CAD* coronary artery disease, *PRECISE* Predicting Bleeding Complications in Patients Undergoing Stent Implantation and Subsequent Dual Antiplatelet Therapy.

### Clinical outcomes

**Figure 1:**
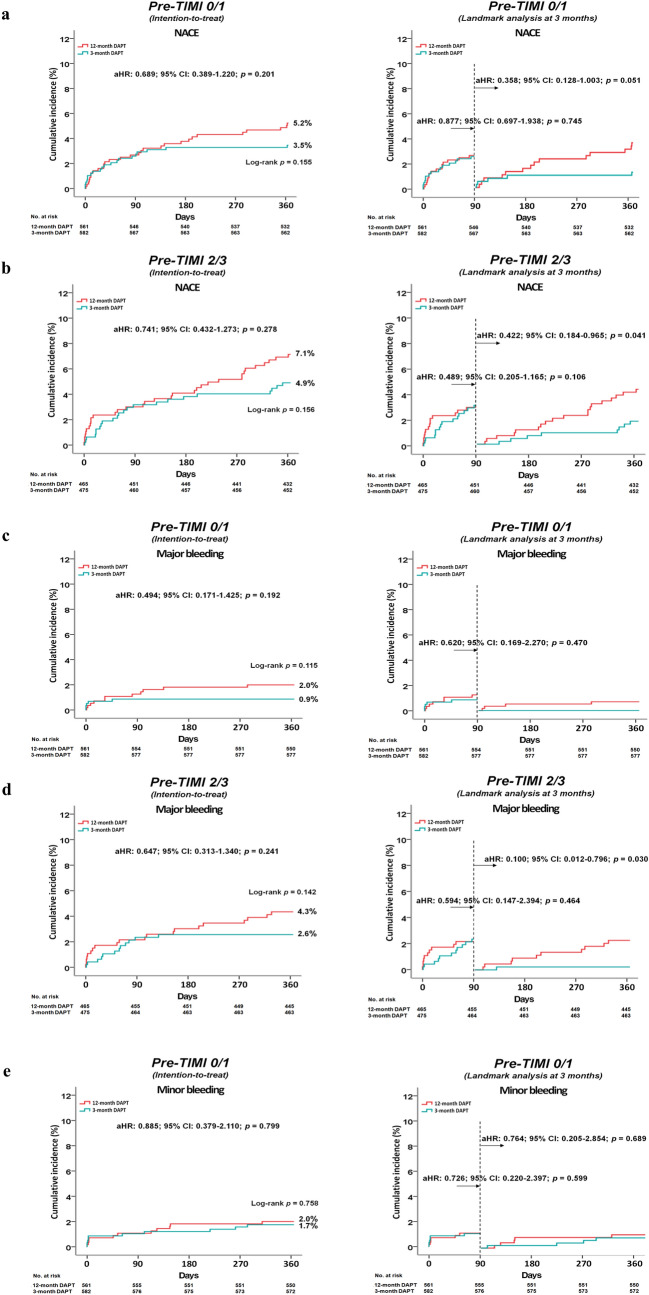

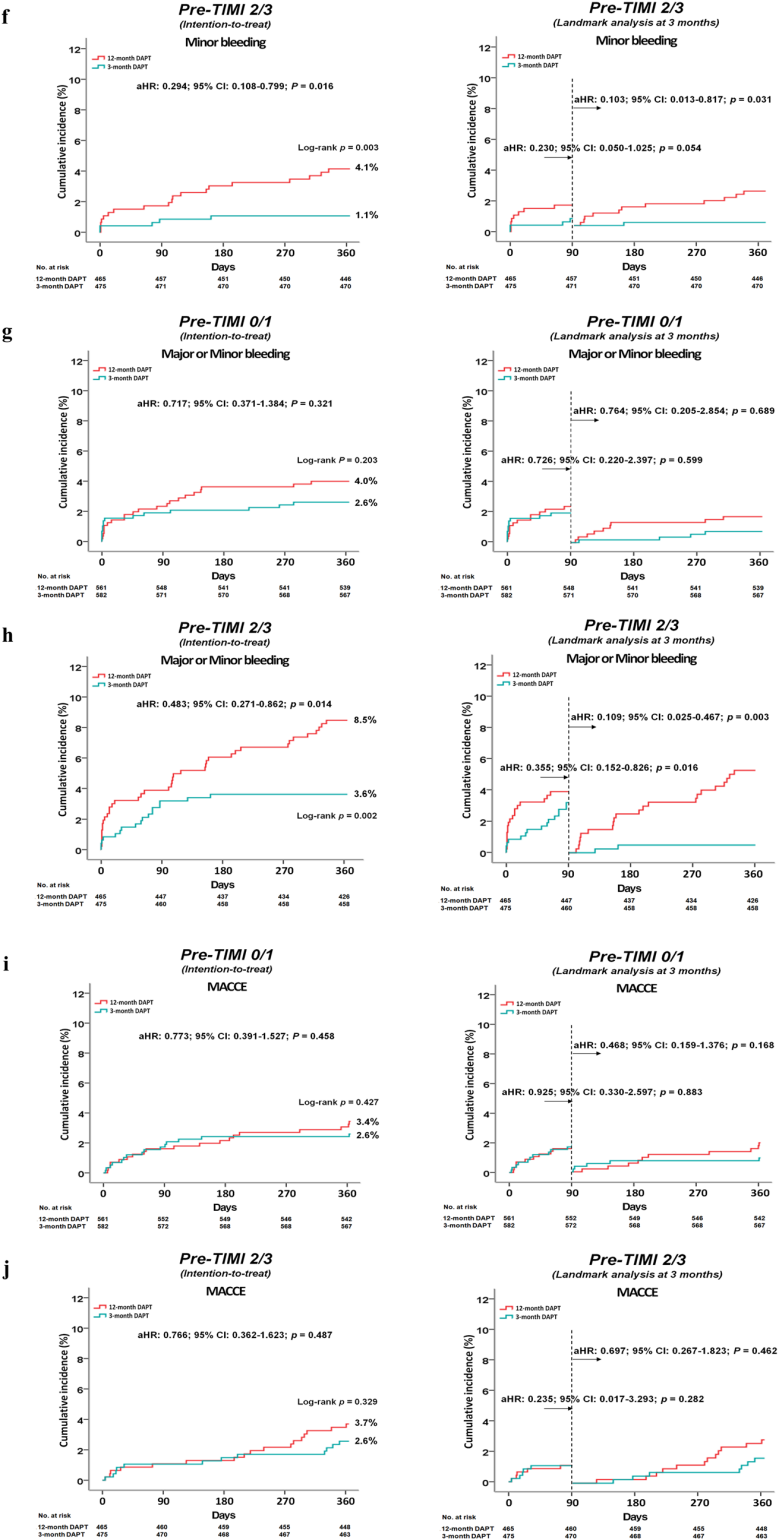
Time-to-event curves for NACE (**a** and **b**), TIMI major bleeding (**c** and **d**), TIMI minor bleeding (**e** and **f**), TIMI major or minor bleeding (**g** and **h**), and MACCE (**i** and **j**) in pre-PCI TIMI flow grade 0/1 (**a**, **c**, **e**, **g**, and **i**) and 2/3 groups (**b**, **d**, **f**, **h**, and **j**).

Clinical outcomes are summarized in Tables 2, 3, and 4, Supplementary materials 7–10, and Fig. 1a–j.

**Table 2:** Clinical outcomes by Kaplan–Meier analysis and Cox-proportional hazard ratio analysis at 1 year.

Pre-PCI TIMI 0/1 (n = 1143)							
Outcomes	Cumulative events (%)			Unadjusted		Adjusted^a^	
	Ticagrelor monotherapy after 3-mo DAPT (n = 582)	Ticagrelor-based 12-mo DAPT group (n = 561)	Log-rank	HR (95% CI)	*p*	HR (95% CI)	*p*
NACE	20 (3.5)	29 (5.2)	0.155	0.663 (0.375–1.172)	0.158	0.689 (0.389–1.220)	0.201
**TIMI bleeding**							
Major	5 (0.9)	11 (2.0)	0.115	0.438 (0.152–1.259)	0.125	0.494 (0.171–1.425)	0.192
Minor	10 (1.7)	11 (2.0)	0.758	0.874 (0.371–2.059)	0.759	0.885 (0.379–2.110)	0.799
Major or minor	15 (2.6)	22 (4.0)	0.203	0.655 (0.340–1.263)	0.207	0.717 (0.371–1.384)	0.321
**MACCE**	15 (2.6)	19 (3.4)	0.427	0.761 (0.387–1.497)	0.428	0.773 (0.391–1.527)	0.458
All-cause death	7 (1.2)	8 (1.4)	0.742	0.884 (0.306–2.326)	0.742	0.949 (0.342–2.630)	0.919
Cardiac death	5 (0.9)	6 (1.1)	0.717	0.803 (0.245–2.632)	0.717	0.863 (0.263–2.833)	0.808
Acute MI	3 (0.5)	4 (0.7)	0.667	0.721 (0.161–3.221)	0.668	0.528 (0.113–2.469)	0.417
TVR	3 (0.5)	4 (0.7)	0.664	0.719 (0.161–3.212)	0.666	0.703 (0.155–3.186)	0.648
ST	3 (0.5)	1 (0.2)	0.334	2.901 (0.302–27.89)	0.356	3.216 (0.333–31.05)	0.313
**Stroke**							
Ischemic	3 (0.5)	4 (0.7)	0.668	0.722 (0.161–3.224)	0.669	0.668 (0.146–3.061)	0.604
Hemorrhagic	0	0	–	–			

Pre-PCI TIMI 2/3 (n = 940)							
Outcomes	Cumulative events (%)			Unadjusted		Adjusted^b^	
	Ticagrelor monotherapy after 3-mo DAPT (n = 475)	Ticagrelor-based 12-mo DAPT group (n = 465)	Log-rank	HR (95% CI)	*p*	HR (95% CI)	*p*
NACE	23 (4.9)	33 (7.1)	0.156	0.682 (0.400–1.161)	0.159	0.741 (0.432–1.273)	0.278
**TIMI bleeding**							
Major	12 (2.6)	20 (4.3)	0.142	0.589 (0.288–1.204)	0.147	0.647 (0.313–1.340)	0.241
Minor	5 (1.1)	19 (4.1)	0.003	0.256 (0.096–0.686)	0.007	0.294 (0.108–0.799)	0.016
Major or minor	17 (3.6)	39 (8.5)	0.002	0.423 (0.239–0.748)	0.003	0.483 (0.271–0.862)	0.014
**MACCE**	12 (2.6)	17 (3.7)	0.329	0.694 (0.331–1.452)	0.332	0.766 (0.362–1.623)	0.487
All-cause death	6 (1.3)	10 (2.2)	0.301	0.590 (0.214–1.622)	0.306	0.700 (0.247–1.985)	0.503
Cardiac death	2 (0.4)	4 (0.9)	0.401	0.490 (0.090–2.677)	0.411	0.391 (0.068–2.254)	0.294
Acute MI	2 (0.4)	5 (1.1)	0.246	0.392 (0.076–2.023)	0.264	0.394 (0.076–2.041)	0.267
TVR	3 (0.7)	3 (0.7)	0.980	0.979 (0.198–4.852)	0.980	0.965 (0.192–4.856)	0.966
ST	2 (0.4)	3 (0.6)	0.638	0.653 (0.109–3.906)	0.640	0.545 (0.085–3.496)	0.522
**Stroke**							
Ischemic	1 (0.2)	0	0.320	–			
Hemorrhagic	1 (0.2)	0	0.322	–			

*Pre-PCI* pre-percutaneous coronary intervention, *TIMI* Thrombolysis In Myocardial Infarction, *DAPT* dual antiplatelet therapy, *HR* hazard ratio, *CI* confidence interval, *NACE* net adverse clinical events, *MACCE* major adverse cardiac and cerebrovascular events, *MI* myocardial infarction, *TVR* target vessel revascularization, *ST* stent thrombosis, *eGFR* estimated glomerular filtration rate, *LVEF* left ventricular ejection fraction, *STEMI* ST-segment elevation myocardial infarction.

^a^Adjusted by age, prior MI, serum creatinine, eGFR, and stent diameter (Supplementary material 15).

^b^Adjusted by age, male, LVEF, hypertension, diabetes mellitus, prior PCI, serum creatinine, and eGFR (Supplementary material 15).

**Table 3:** Clinical outcomes between pre-PCI TIMI 0/1 and 2/3 groups according to 3-month or 12-month DAPT strategies.

Ticagrelor monotherapy after 3-mo DAPT (n = 1057)							
Outcomes	Cumulative events (%)			Unadjusted		Adjusted^a^	
	Pre-PCI TIMI 0/1 (n = 582)	Pre-PCI TIMI 2/3 (n = 475)	Log-rank	HR (95% CI)	*p* value	HR (95% CI)	*p* value
NACE	20 (3.5)	23 (4.9)	0.257	0.708 (0.389–1.289)	0.259	0.729 (0.384–1.384)	0.333
**TIMI bleeding**							
Major	5 (0.9)	12 (2.6)	0.033	0.339 (0.120–0.963)	0.042	0.301 (0.090–1.003)	0.051
Minor	10 (1.7)	5 (1.1)	0.364	1.635 (0.559–4.784)	0.369	2.129 (0.694–6.533)	0.187
Major or minor	15 (2.6)	17 (3.6)	0.349	0.719 (0.359–1.439)	0.351	0.773 (0.396–1.622)	0.496
**MACCE**	15 (2.6)	12 (2.6)	0.952	1.024 (0.479–2.187)	0.952	1.151 (0.513–2.580)	0.733
All-cause death	7 (1.2)	6 (1.3)	0.935	0.956 (0.321–2.844)	0.935	1.261 (0.391–4.066)	0.698
Cardiac death	5 (0.9)	2 (0.4)	0.382	2.047 (0.397–10.55)	0.392	2.263 (0.423–12.12)	0.340
Acute MI	3 (0.5)	2 (0.4)	0.822	1.288 (0.205–7.349)	0.822	2.297 (0.370–14.25)	0.372
TVR	3 (0.5)	3 (0.7)	0.802	0.815 (0.165–4.039)	0.802	0.816 (0.132–5.064)	0.827
ST	3 (0.5)	2 (0.4)	0.366	1.261 (0.391–4.066)	0.698	1.243 (0.198–7.796)	0.817
**Stroke**							
Ischemic	3 (0.5)	1 (0.2)	0.422	2.451 (0.256–23.57)	0.437	2.544 (0.229–28.27)	0.401
Hemorrhagic	0	1 (0.2)	0.270	–	–	–	–

Ticagrelor-based 12-mo DAPT group (n = 1026)							
Outcomes	Cumulative events (%)			Unadjusted		Adjusted^b^	
	Pre-PCI TIMI 0/1 (n = 561)	Pre-PCI TIMI 2/3 (n = 465)	Log-rank	HR (95% CI)	*p* value	HR (95% CI)	*p* value
NACE	29 (5.2)	33 (7.1)	0.213	0.729 (0.443–1.201)	0.215	0.863 (0.513–1.451)	0.578
**TIMI bleeding**							
Major	11 (2.0)	20 (4.3)	0.032	0.456 (0.219–0.952)	0.037	0.519 (0.241–1.116)	0.093
Minor	11 (2.0)	19 (4.1)	0.048	0.481 (0.229–1.010)	0.053	0.526 (0.244–1.132)	0.100
Major or minor	22 (4.0)	39 (8.5)	0.003	0.465 (0.276–0.784)	0.004	0.514 (0.299–0.884)	0.016
**MACCE**	19 (3.4)	17 (3.7)	0.844	0.936 (0.487–1.802)	0.844	1.207 (0.609–2.390)	0.590
All-cause death	8 (1.4)	10 (2.2)	0.392	0.668 (0.264–1.692)	0.395	0.985 (0.373–2.605)	0.976
Cardiac death	6 (1.1)	4 (0.9)	0.727	1.252 (0.353–4.436)	0.728	1.712 (0.461–6.367)	0.422
Acute MI	4 (0.7)	5 (1.1)	0.544	0.667 (0.179–2.485)	0.547	0.866 (0.215–3.480)	0.839
TVR	4 (0.7)	3 (0.7)	0.890	1.111 (0.249–4.964)	0.890	1.049 (0.223–4.924)	0.952
ST	1 (0.2)	3 (0.6)	0.236	0.278 (0.029–2.674)	0.268	0.346 (0.033–3.617)	0.376
**Stroke**							
Ischemic	4 (0.7)	0	0.066	–	–	–	–
Hemorrhagic	0	0	–	–	–	–	–

*Pre-PCI* pre-percutaneous coronary intervention, *TIMI* Thrombolysis In Myocardial Infarction, *DAPT* dual antiplatelet therapy, *HR* hazard ratio, *CI* confidence interval, *NACE* net adverse clinical events, *MACCE* major adverse cardiac and cerebrovascular events, *MI* myocardial infarction, *TVR* target vessel revascularization, *ST* stent thrombosis, *LVEF* left ventricular ejection fraction, *STEMI* ST-segment elevation myocardial infarction, *LM* left main coronary artery, *ACE* angiotensin converting enzyme, *CCB* calcium channel blocker.

^a^Adjusted by age, LVEF, diabetes mellitus, white blood cell, hemoglobin, STEMI, LM, and single-vessel disease (Supplementary material 16).

^b^Adjusted by age, male, hypertension, diabetes mellitus, prior MI, prior PCI, hemoglobin, STEMI, beta-blocker, ACE inhibitor, CCB, transfemoral approach, and stent diameter (Supplementary material 16).

**Table 4:** Interaction between pre-PCI TIMI during an index PCI (pre-PCI TIMI 0/1 vs. 2/3) and the duration of DAPT (3-mo DAPT vs. 12-mo DAPT) for clinical outcomes.

Outcomes	Interaction *p* value (Intention-to-treat)		Interaction *p* value (As-treated)	
	Unadjusted	Adjusted^a^	Unadjusted	Adjusted^a^
NACE	0.043	0.476	0.040	0.395
**TIMI bleeding**				
Major	0.011	0.329	0.014	0.400
Minor	0.394	0.714	0.291	0.730
Major or minor	0.012	0.653	0.010	0.650
**MACCE**	0.473	0.836	0.396	0.716
All-cause death	0.512	0.505	0.583	0.909
Cardiac death	0.887	0.108	0.820	0.540
Acute MI	0.391	0.571	0.296	0.796
TVR	0.695	0.529	0.353	0.663
ST	0.714	0.532	0.667	0.267
**Stroke**				
Ischemic	0.546	0.622	0.505	0.733
Hemorrhagic	0.710	0.862	0.715	0.904

^a^Adjusted for variables that showed differences with *p* < 0.05 (age, male, LVEF, hypertension, diabetes mellitus, prior MI, prior PCI, serum creatinine, eGFR, STEMI, beta-blocker, CCB, and stent diameter) (Supplementary material 15)  between the pre-PCI TIMI 0/1 and pre-PCI 2/3 groups.

*Pre-PCI* pre-percutaneous coronary intervention, *TIMI* Thrombolysis In Myocardial Infarction, *DAPT* dual antiplatelet therapy, *3-mo* 3-month, *12-mo* 12-month, *NACE* net adverse clinical events, *MACCE* major adverse cardiac and cerebrovascular events, *MI* myocardial infarction, *TVR* target vessel revascularization, *ST* stent thrombosis, *LVEF* left ventricular ejection fraction, *eGFR* estimated glomerular filtration rate, STEMI ST-segment elevation myocardial infarction, *CCB* calcium channel blocker.

**Figure 2:**
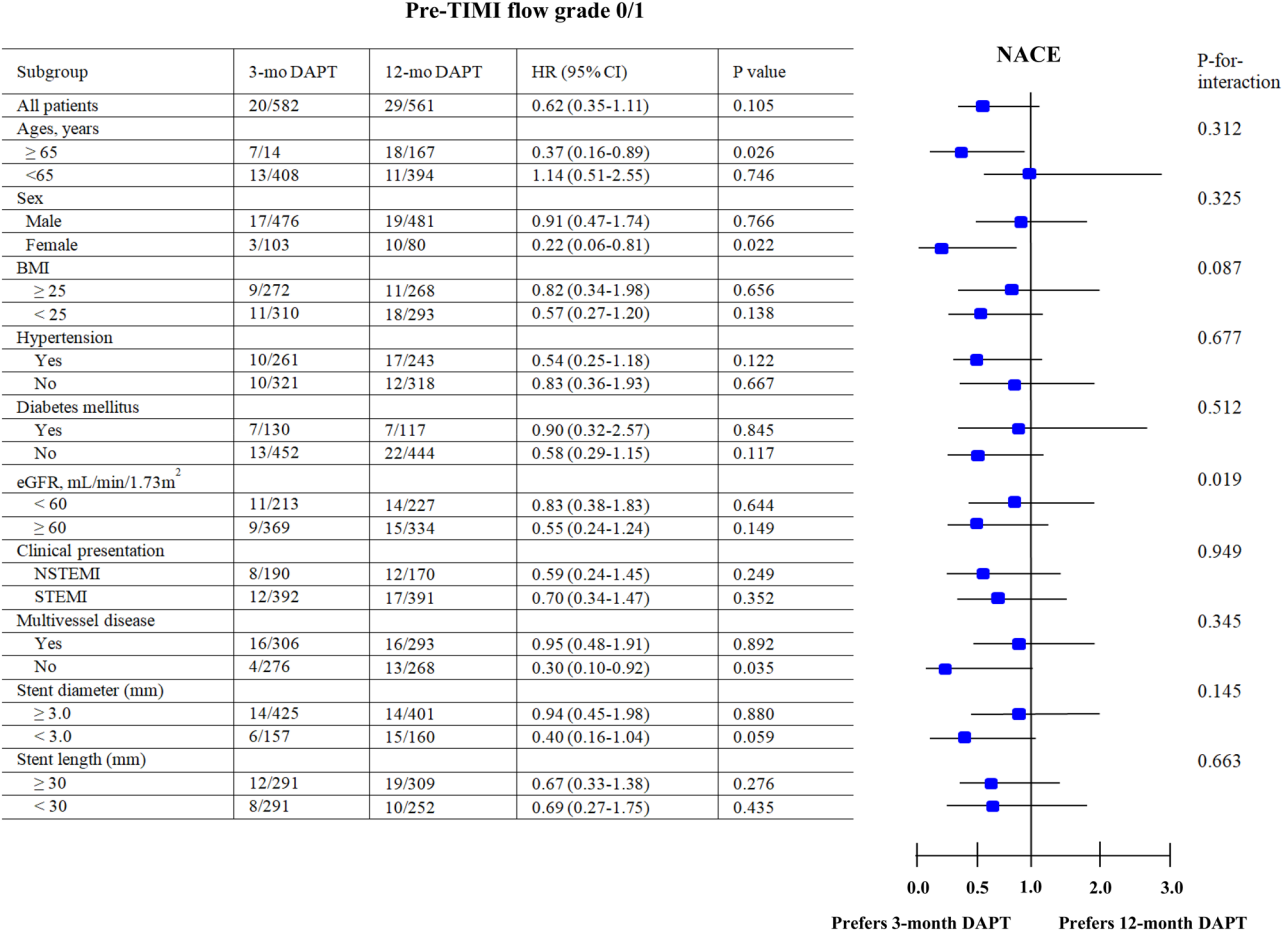
Subgroup analysis for NACE in pre-TIMI flow grade 0/1 group. * NACE* net adverse clinical event,* Pre-PCI* pre-percutaneous coronary intervention,* TIMI* Thrombolysis In Myocardial Infarction,* 3-mo DAPT*, ticagrelor monotherapy after 3-month dual antiplatelet therapy,* 12-mo* DAPT ticagrelor-based 12-month dual antiplatelet therapy,* HR* hazard ratio,* CI* confidence interval,* BMI* body mass index,* eGFR* estimated glomerular filtration rate,* NSTEMI* non-ST segment elevation myocardial infarction,* STEMI* ST segment elevation myocardial infarction.

### Net adverse clinical events (NACE)

Table 2 shows clinical outcomes by Kaplan–Meier analysis and Cox-proportional hazard ratio analysis. In patients in both pre-TIMI 0/1 and 2/3 groups, the occurrence of NACE was not significantly different between the 3-mo and 12-mo DAPT groups (adjusted hazard ratio [aHR]: 0.689; 95% confidence interval [CI]: 0.389–1.220; *p* = 0.201; and aHR: 0.741; 95% CI: 0.432–1.273; *p* = 0.278, respectively) (Fig. 1a,b). In patients in both as-treated pre-TIMI 0/1 and 2/3 groups, the occurrence of NACE was not significantly different between 3-mo and 12-mo DAPT groups (Supplementary materials 7 and 8). On 3-mo landmark analyses between the 3-mo and 12-mo groups (Supplementary material 9), although the occurrence of NACE was not significantly different between 3-mo and 12-mo DAPT (aHR: 0.358; 95% CI: 0.128–1.003; *p* = 0.051) in pre-TIMI 0/1 group, it was significantly higher in the 12-mo DAPT group than in 3-mo DAPT group in the pre-TIMI 2/3 group (aHR: 0.422; 95% CI: 0.184–0.965; *p* = 0.041).

### Thrombolysis in myocardial infarction (TIMI) bleedings

In patients with pre-TIMI 0/1, the occurrence of TIMI major, minor, and major or minor bleedings (Table 2, Fig. 1c,e,g) were not significantly different between the 3-mo and 12-mo DAPT groups. These results were repeated in patients in the as-treated group and on the 3-mo landmark analysis. In patients with pre-TIMI 2/3, although the occurrence of TIMI major bleeding was similar between the 3-mo and 12-mo DAPT groups (Table 2 and Fig. 1d), the occurrence of TIMI minor bleeding (aHR: 0.294; 95% CI: 0.108–0.799; *p* = 0.016) and TIMI major or minor bleeding (aHR: 0.483; 95% CI: 0.271–0.862; *p* = 0.014) were significantly higher in the 12-mo DAPT group than in the 3-mo DAPT group (Table 2, Fig. 1f,h). These results were repeated in patients in the as-treated group. However, on 3-mo landmark analyses between the 3-mo and 12-mo groups (Supplementary material 9), the occurrence of TIMI major, minor, and major or minor bleeding in patients in the pre-TIMI 2/3 group was significantly higher in the 12-mo DAPT group than in the 3-mo DAPT group (aHR: 0.100; 95% CI: 0.012–0.796; *p* = 0.030, aHR: 0.103; 95% CI: 0.013–0.817; *p* = 0.031, and aHR: 0.109; 95% CI: 0.025–0.467; *p* = 0.003, respectively).

### Major adverse cardiac and cerebrovascular events (MACCE)

The occurrence of MACCE was not significantly different between the 3-mo and 12-mo DAPT groups in patients in both pre-TIMI 0/1 (aHR: 0.773; 95% CI: 0.391–1.527; *p* = 0.458, Table 2, Fig. 1i) and 2/3 groups (aHR: 0.766; 95% CI: 0.362–1.623; *p* = 0.487, Table 2, Fig. 1j). These results were repeated in patients in the as-treated group and on the 3-mo landmark analysis.

### Subgroup analyses

**Figure 3:**
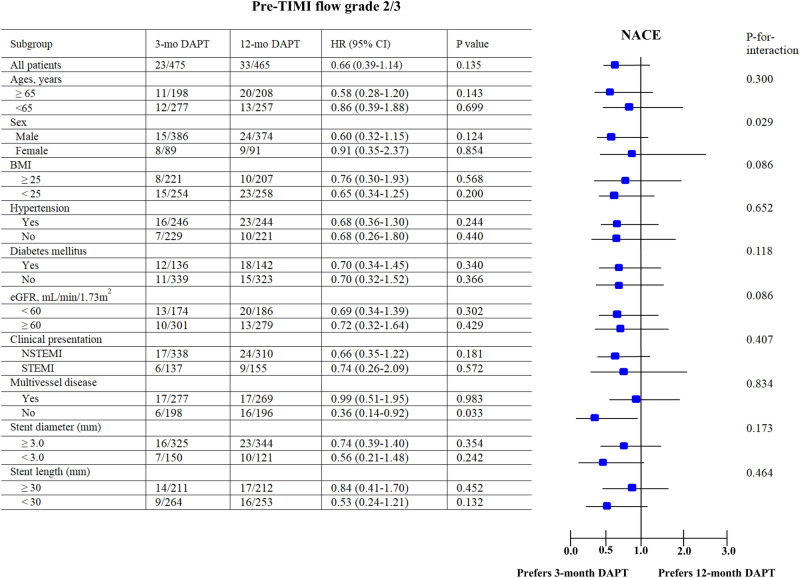
Subgroup analysis for NACE in pre-TIMI flow grade 2/3 group. * NACE* net adverse clinical event,* Pre-PCI* pre-percutaneous coronary intervention,* TIMI* Thrombolysis In Myocardial Infarction,* 3-mo DAPT*, ticagrelor monotherapy after 3-month dual antiplatelet therapy,* 12-mo* DAPT ticagrelor-based 12-month dual antiplatelet therapy,* HR* hazard ratio,* CI* confidence interval,* BMI* body mass index,* eGFR* estimated glomerular filtration rate,* NSTEMI* non-ST segment elevation myocardial infarction,* STEMI* ST segment elevation myocardial infarction.

Subgroup analyses for NACE are shown in Figs. 2 and 3. In patients with pre-TIMI 0/1 (Fig. 2) and old age (≥ 65 years, HR: 0.37; 0.16–0.89; *p* = 0.026), female (HR: 0.22; 0.06–0.81; *p* = 0.022), those with single-vessel disease (HR: 0.30; 0.10–0.92; *p* = 0.035); and in patients in the pre-TIMI 2/3 group (Fig. 3) with single-vessel disease (HR: 0.36; 0.14–0.92; *p* = 0.033), 3-mo DAPT showed better outcomes over 12-mo DAPT in this study.

**Figure 4:**
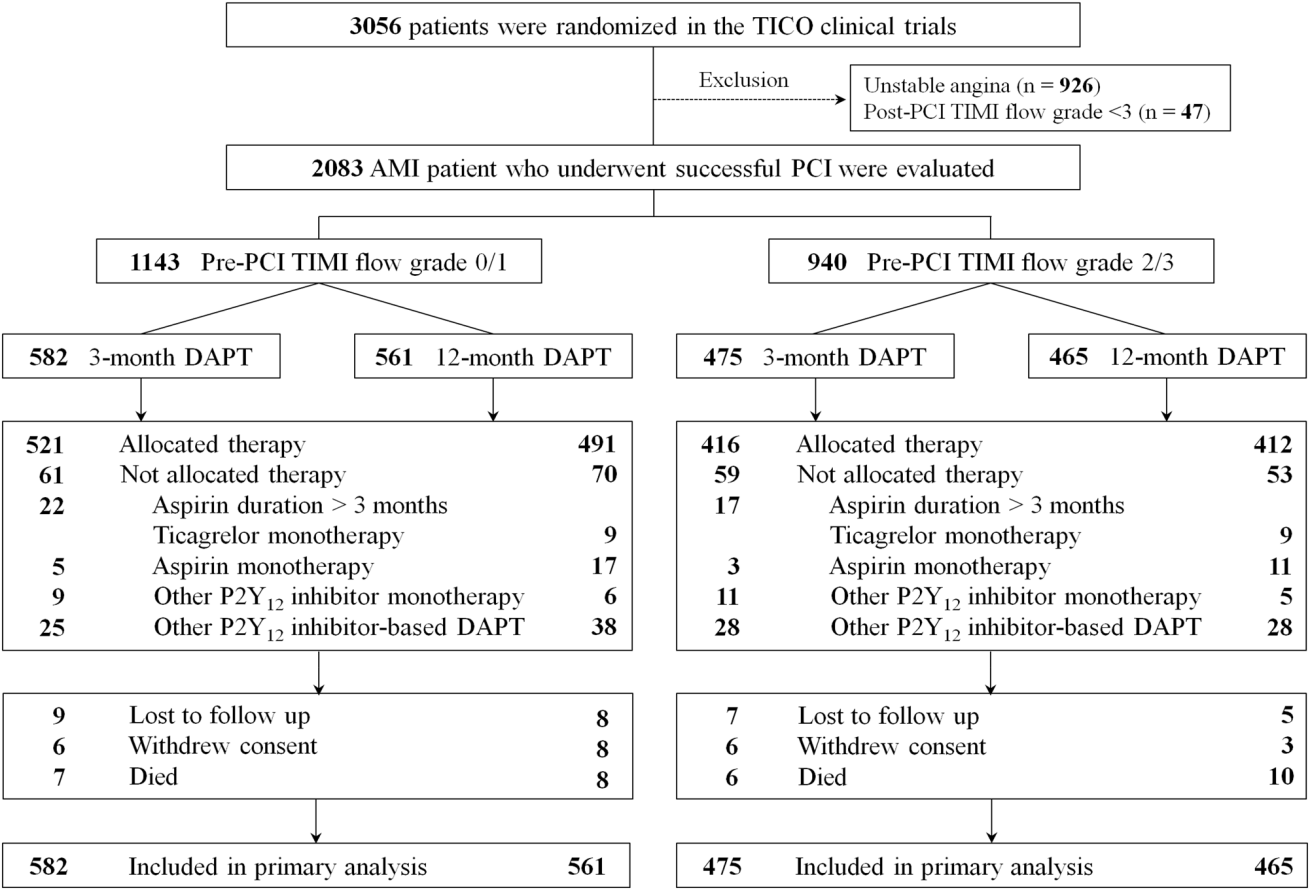
Flowchart.* TICO* Ticagrelor Monotherapy After 3 Months in the Patients Treated With New Generation Sirolimus-eluting Stent for Acute Coronary Syndrome,* Pre-PCI* pre-percutaneous coronary intervention,* TIMI* Thrombolysis In Myocardial Infarction,* DAPT* dual antiplatelet therapy.

### Independent predictors for NACE

In Supplementary material 11, after multivariate analysis of patients with pre-TIMI 0/1, age, prior MI, eGFR, and diameter of deployed stents were independent predictors for NACE. In patients with pre-TIMI 2/3, diabetes mellitus was an independent predictor of NACE in this study.

### Clinical outcomes between pre-TIMI 0/1 and 2/3 groups according to 3-month or 12-month DAPT strategies

In Table 3, in patients with 3-mo DAPT, the occurrence of NACE, TIMI major, minor, and major or minor bleeding was similar between the pre-TIMI 0/1 group and the pre-TIMI 2/3 group after adjustment. However, in patients with 12-mo DAPT, the occurrence of TIMI major or minor bleeding was significantly higher in the pre-TIMI 2/3 group than in the pre-TIMI 0/1 group (aHR: 0.514; 95% CI: 0.299–0.884; *p* = 0.016).

### ST-segment elevation versus non-ST-segment elevation myocardial infarction (STEMI vs. NSTEMI)

The comparison of clinical outcomes between STEMI and NSTEMI is summarized in Supplementary material 12. After adjustment, in patients with both pre-TIMI 0/1 and 2/3, the occurrence of NACE, TIMI bleedings (major, minor, and major or minor), and MACCE were not significantly different between STEMI and NSTEMI. Supplementary material 13 shows univariate analysis for NACE according to the pre-TIMI in comparing STEMI and NSTEMI.

### Interaction between pre-TIMI during an index PCI and the duration of DAPT

Table 4 shows the interaction between pre-TIMI during an index PCI (pre-TIMI 0/1 vs. 2/3) and the duration of DAPT (3-mo or 12-mo DAPT). There were no significant interactions between the different pre-PCI TIMI during an index PCI and the duration of DAPT after adjustment.

## Discussion

The TIMI flow grade is a traditional method for assessing coronary blood flow^11^. Previous studies have shown that various pro-thrombotic markers including platelet count, reactivity, and mean platelet volume were associated with patency of the infarct-related artery in patients with STEMI, before primary PCI^12,13^. Moreover, in the CADILLAC (Controlled Abciximab and Device Investigation to Lower Late Angioplasty Complications) and HORIZONS-AMI (Harmonizing Outcomes with RevasculariZatiON and Stents in Acute Myocardial Infarction) Trials, pre-TIMI 3 was an important independent predictor of 1-year survival^14^. The main findings of this study were as follows: (1) in patients with pre-TIMI 0/1, the occurrence of NACE, TIMI bleedings (major, minor, and major or minor), and MACCE (all-cause death, cardiac death, MI, ST, and stroke) were not significantly different between the 3-mo and 12-mo DAPT groups. (2) In patients with pre-TIMI 2/3, the occurrence of TIMI minor bleeding and major or minor bleeding were significantly higher in the 12-mo DAPT group than in the 3-mo DAPT group. Moreover, on 3-mo landmark analyses between 3-mo and 12-mo, the occurrence of TIMI major bleeding was significantly higher in the 12-mo DAPT group than in the 3-mo DAPT group. However, the occurrence of MACCE was similar between the 3-mo and 12-mo DAPT groups. (3) The occurrence of NACE, TIMI bleeding, and MACCE were not significantly different between STEMI and NSTEMI.

The present data indicate that pre-TIMI 0/1 is present in 54.8% (STEMI, 68.5%, vs. NSTEMI, 31.5%) and pre-TIMI 2/3 is present in 45.2% (STEMI, 31.1%, vs. NSTEMI, 68.9%). The ratio STEMI/NSTEMI is at the complete opposite between the pre-TIMI 0/1 and 2/3 groups. However, these results are comparable with Bailleul et al. study^15^. Although the proportions of STEMI and NSTEMI in patients with pre-TIMI 0/1 or 2/3 were different, these proportions were not significantly different between the 3-mo DAPT or 12-mo DAPT groups (Table 1). Especially, in Table 3, STEMI was included in the multivariate analysis as a significant variable with other variables. The occurrence of TIMI major or minor bleeding was significantly in the pre-TIMI 2/3 group than in the pre-TIMI 0/1 group, similar to the results in Table 2. In addition, as shown in Supplementary material 10, in patients with both pre-TIMI 0/1 and 2/3, the occurrence of NACE, TIMI bleeding (major, minor, and major or minor), and MACCE were similar between STEMI and NSTEMI.

A ruptured, eroded, or protruding calcified atherosclerotic plaque could trigger local thrombosis, which is a critical step in the pathogenesis of AMI^16^. To date, it remains unclear why some plaques lead to STEMI with poor pre-TIMI but others do not^17^. Compared to pre-TIMI 0/1, which has prolonged ischemia and late reperfusion, can impair endothelial function, and cause myocardial tissue edema, pre-TIMI 2/3 would have shorter ischemic time and less myocardial damage^17^. In a state of endothelial dysfunction, disruption of the balance between anti-thrombosis and pro-thrombosis can lead to increased platelet aggregation^18^. More recently, Bauer et al.^19^ reported that, after adjustment, definite stent thrombosis (ST) occurred only in patients with pre-TIMI 0/1 in their ATLANTIC (Administration of Ticagrelor in the Cath Lab or in the Ambulance for New ST Elevation Myocardial Infarction to Open the Coronary Artery) sub-study. Moreover, they showed that prehospital administration of ticagrelor was less effective in patients with pre-TIMI 0/1 than those with pre-TIMI 2/3 (0.3% vs. 1.3%, *p* < 0.05). Hence, it could be assumed that on treatment with 3-mo or 12-mo DAPT, the major clinical outcomes could be influenced by pre-TIMI. However, the effect of pre-TIMI on bleeding and cardiovascular events after ticagrelor-based 3-mo or 12-mo DAPT in patients with AMI has not been reported. Thus, this paper may be considered as the first report focused on this perspective.

In our study, compared to the patients with pre-TIMI 0/1, the occurrences of TIMI minor bleeding and major or minor bleeding were significantly higher in the 12-mo DAPT group than in the 3-mo DAPT group, in patients with pre-TIMI 2/3. In addition, on 3-mo landmark analyses between 3-mo and 12-mo, the occurrence of NACE and TIMI major, minor, and major and minor bleeding were also higher in the 12-mo DAPT group than in the 3-mo DAPT group. Because of the absence of previous reports, it could be difficult to provide comparative results between our and previous reports. However, based on our results, it could be considered that the beneficial effects of 3-mo DAPT over 12-mo DAPT in reducing bleeding events are mainly determined by pre-TIMI 2/3 rather than by pre-TIMI 0/1. However, in patients with 12-mo DAPT, the mean age, the number of hypertensive and diabetic patients, and patients with prior history of PCI were significantly higher in the pre-TIMI 2/3 group than in the pre-TIMI 0/1 group. In this study, to adjust the diverse variables, multivariate analysis was performed. But, it could be speculated that these baseline characteristics may play an important role in explaining this higher TIMI major or minor bleeding. Despite the possible benefit of DAPT in reducing ischemic events of infarction, it may be considered that no compound can enter an ischemic no-flow area of myocardium, especially if the culprit coronary artery in totally occlude^7^. Hence, in patients with pre-TIMI 0/1, the occurrence of NACE, TIMI bleeding, and MACCE would not be significantly different between the 3-mo or 12-mo DAPT groups. In contrast, patients with pre-TIMI 2/3 treated with 12-mo DAPT showed a higher incidence of bleeding tendency than those with 3-mo DAPT without showing increased incidences of ischemic events. According to the subgroup analysis (Figs. 2 and 3), in both pre-TIMI 0/1 and 2/3, and in patients with single-vessel disease, 3-mo DAPT may be preferred over 12-mo DAPT to reduce NACE in this study.

In the FAST-MI (French Registry of Acute ST-Elevation or Non-ST-Elevation Myocardial infarction) study, after 2010, there was no further mortality gain was founded in patients with STEMI with reperfusion therapy or in patients with NSTEMI, regardless of performing PCI^20^. Moreover, there are some debates regarding the long-term prognosis between STEMI and NSTEMI^21–23^, the occurrence of NACE, TIMI bleeding, and MACCE were not significantly different between these two groups both in pre-TIMI 0/1 and 2/3 groups (Supplementary material 12) in our study and our results were consistent with those of Montalescot’s findings^23^.

Based on our results, considering pre-TIMI may be helpful to understand more accurately the comparative outcomes between short and standard 12-mo DAPT in patients with AMI receiving new-generation DES.

This study has some limitations. First, although the TICO trial was an open-label trial, this trial was not a placebo-controlled study. Therefore, drug adherence was not monitored. Second, the lower-than-expected rate of adverse events in the main TICO trial could be a limitation in this study. Therefore, caution regarding the interpretation of our study results is needed. Third, because the ultrathin bioresorbable polymer sirolimuls-eluting stent (Orsiro; BIOTRONIK, Buelach, Switzerland) was deployed in the whole study population in this study, diverse results that would reflect many other kinds of new-generation DES that are currently in use were not included in this study. Fourth, because platelet function tests (e.g., P2Y_12_ reaction unit) and aspirin reaction unit, mean platelet volume, and myocardial blush grade were not monitored before, during, and after PCI in the TICO trial, we could not provide this information. Fifth, because the use of intravascular ultrasound, optical coherence tomography, and fractional flow reserve (FFR) were not mandatory tools for treatment in the main TICO trial, we could not provide results according to the use of these imaging and functional testing tools for the lesions. Finally, even if pre-TIMI is easy and inexpensive, it could be a suboptimal, incomplete measure of myocardial perfusion. Moreover, although the TIMI flow grade is often used to evaluate blood flow during acute coronary occlusion and/or reperfusion, as a more instructional reference and more relevant indicators, such as FFR, should be considered to give a more accurate evaluation.

In conclusion, our results suggest that the higher bleeding tendency in 12-mo DAPT compared with 3-mo DAPT was more obvious in patients with pre-TIMI 2/3 than in those with pre-TIMI 0/1. However, more studies are warranted to confirm these results.

## Methods

### Study design

A total of 3056 participants from the TICO randomized clinical trial (ClinicalTrials.gov Identifier: NCT02494895; First registration: 10/07/2015)^4,24^ were evaluated in this study. The TICO trial was an investigator-initiated, multicenter, randomized, unblinded trial conducted at 38 centers in South Korea. Briefly, after PCI, patients were randomly assigned in a 1:1 ratio to receive ticagrelor monotherapy after 3-mo DAPT or ticagrelor-based 12-mo DAPT. A web-response permuted-block randomization was used, and the allocation sequence was computer generated by an external programmer. If patients were not taking aspirin or ticagrelor at the time of PCI, loading doses of aspirin (300 mg) and ticagrelor (180 mg) were administered. A single aspirin dose (100 mg per day) and 2 ticagrelor (180 mg per day) were maintained. After 3-mo of DAPT, aspirin use was continued in patients who were randomized to receive 12-mo ticagrelor-based DAPT group. The concomitant use of other antiplatelet agents or anticoagulants was not allowed. Other medical treatments were left to physician discretion^4^. The main aim of the TICO trial was to estimate the validity of ticagrelor monotherapy after 3-mo of DAPT compared to 12-mo of aspirin and ticagrelor DAPT, with respect to bleeding outcomes and MACCE, in patients with acute coronary syndrome (ACS) who received the Orsiro® (BIOTRONIK, Buelach, Switzerland) stent.

### Study population

Key exclusion criteria included increased risk of bleeding due to prior hemorrhagic stroke, traumatic brain injury or brain surgery within the past 6 months, internal bleeding within the past 6 weeks, need of oral anticoagulation therapy, and anemia (hemoglobin ≤ 8 g/dL)^4^. The full inclusion and exclusion criteria are listed in Supplementary material 14. Additionally, patients with unstable angina (n = 926, 30.3%) and those with post-PCI TIMI flow grade < 3 (n = 47, 1.5%) were excluded. During a 12-mo follow-up period, 17 patients in the pre-TIMI 0/1 group (3-mo DAPT, n = 9; 12-mo DAPT, n = 8) and 12 patients in the pre-TIMI 2/3 group (3-mo DAPT, n = 7; 12-mo DAPT, n = 5) were lost to follow-up. Participants who withdrew the consent (pre-TIMI 0/1 group, n = 14 [3-mo DAPT, n = 6; 12-mo DAPT, n = 8]; pre-TIMI 2/3 group, n = 9 [3-mo DAPT, n = 6; 12-mo DAPT, n = 3]) or those who died (pre-TIMI 0/1 group, n = 15 [3-mo DAPT, n = 7; 12-mo DAPT, n = 8]; pre-TIMI 2/3 group, n = 16 [3-mo DAPT, n = 6; 12-mo DAPT, n = 10]) were also excluded. Hence, a total of 2083 AMI patients were finally included. The patients were classified into pre-PCI TIMI flow grade 0/1 (pre-TIMI 0/1, n = 1143, 54.9%) and pre-TIMI 2/3 (n = 940, 45.1%) groups. Thereafter, the pre-TIMI 0/1 group was further divided into the ticagrelor monotherapy after 3-mo DAPT (3-mo DAPT, n = 582, 50.9%) group and the ticagrelor-based 12-mo DAPT (12-mo DAPT, n = 561, 49.1%) group. The pre-TIMI 2/3 group was also divided into the 3-mo (n = 475, 50.5%) and 12-mo (n = 465, 49.5%) DAPT groups (Fig. 4). The study protocol was approved by the Institutional Review Board (IRB) of each participating center and the Kangwon National University IRB (No. KNUH-2021-02-014), and was conducted in compliance with the ethical standards of the Declaration of Helsinki. Informed written consent was obtained from all patients prior to their inclusion in the study.

### PCI procedure and medical treatment

Diagnostic coronary angiography and PCI were performed using standard techniques^25^. If the patient was not taking aspirin or ticagrelor at the time of PCI, a loading dose of aspirin (300 mg) and ticagrelor (180 mg) were administered before PCI. Thereafter, 100 mg of aspirin per day and 90 mg of ticagrelor twice per day were prescribed as daily maintenance therapy^4^. After 3-mo DAPT consisting of aspirin and ticagrelor, aspirin was discontinued in the ticagrelor monotherapy group and continued in the 12-mo DAPT group^4^.

### Study endpoints and definitions

The primary outcome was the occurrence of a NACE, defined as a composite of TIMI major bleeding and MACCE within 12 months of index PCI. The second outcome was the occurrence of TIMI major, minor, and major or minor bleeding and the occurrence of individual components of MACCE, defined as all-cause death, cardiac death (CD), myocardial infarction (MI), target vessel revascularization (TVR), ST, and stroke. Major bleeding was defined according to the TIMI criteria: intracranial bleeding, hemorrhage with a hemoglobin decrease of at least 5 g/dL, or fatal bleeding that caused death within 7 days^4,26^. Definitions of CD, MI, TVR, ST, and stroke have already been published^4^. In case of NSTEMI, culprit vessel was evaluated by coronary angiographic findings, 12-lead electrocardiogram, two-dimensional echocardiogram, and noninvasive stress test^27,28^. A successful PCI was defined as a residual stenosis of < 30% and TIMI flow grade 3 for the infarct-related artery after the procedure. All baseline and procedural angiographic images including TIMI flow grade of the enrolled patients were centrally collected, and quantitative and qualitative analyses were independently performed in the central angiographic core laboratory (Cardiovascular Research Institute, Severance Cardiovascular Hospital, Seoul, South Korea). Moreover, the PRECISE-DAPT (Predicting Bleeding Complications in Patients Undergoing Stent Implantation and Subsequent Dual Antiplatelet Therapy) score was assessed using an online calculator (http://www.precisedaptscore.com) with 5 variables (age, creatine clearance, hemoglobin, white blood cell count, and previous spontaneous bleeding)^29^. Adverse events were centrally collected, and any document that could lead to unblinding of treatment assignment was obliterated before submission to the clinical event committee. Outcomes were categorized according to predefined criteria by an independent clinical event committee blinded to the treatment assignments and primary results of the trial^4^.

### Statistical analysis

Primary analyses of this study were performed in an intention-to-treat manner. Pre-specified 3-month landmark analyses were performed. Post-hoc analyses were performed for the as-treated population regarding the actual treatments received. Categorical data were reported as numbers and percentages, and they were compared using the chi-square test or Fisher’s exact test, as appropriate. Continuous variables were expressed as mean ± standard deviation, and were compared using the Student’s t-test. Various clinical outcomes were estimated using the Kaplan–Meier method, and intergroup differences were compared using the log-rank test. To determine meaningful variables, all variables with *p* < 0.1 and known conventional risk factors for poor outcomes in the AMI population were considered potential confounding factors and were included in the univariate analysis (Supplementary materials 15 and 16). Variables with *p* < 0.05 were included in the multivariate analysis model. For all analyses, a two-sided *p* value < 0.05 was considered statistically significant. All statistical analyses were performed using SPSS software version 20 (IBM, Armonk, NY, USA).

## Supplementary Information

Supplementary Information 1.

## Data Availability

Data is contained with the article or supplementary material.
