# Supplementary material for: Impact of preprocedural coronary flow grade on duration of dual antiplatelet therapy in acute myocardial infarction
Source: Sci Rep. 2021 Jun 3;11:11735. doi: 10.1038/s41598-021-91130-5 (PMC8175426; doi:10.1038/s41598-021-91130-5)
Supplement: Supplementary file 1 — Supplementary Information 1. [file 41598_2021_91130_MOESM1_ESM.docx]

**Supplementary Appendix**

**Impact of preprocedural coronary flow grade on duration of dual antiplatelet therapy in acute myocardial infarction**

**Short title:** Pre-PCI TIMI flow grade and DAPT

Yong Hoon Kim^1,3^, Ae-Young Her^1,3^, Byeong-Keuk Kim^2^, Sung-Jin Hong^2^, Chul-Min Ahn^2^, Jung-Sun Kim^2^, Young-Guk Ko^2^, Donghoon Choi^2^, Myeong-Ki Hong^2^ & Yangsoo Jang^2^

**Supplementary Online Contents**

**Supplementary material 1.** Antiplatelet therapy during the study period.

**Supplementary material 2.** Causes of non-adherence to the allocated treatment.

**Supplementary material 3.** Medications during the study period.

**Supplementary material 4.** Baseline characteristics according to 3-month or 12-month DAPT strategies.

**Supplementary material 5.** Baseline characteristics for As-treated population.

**Supplementary material 6.** Baseline characteristics for As-treated population according to 3-month or 12-month DAPT strategies.

**Supplementary material 7.** Clinical outcomes of the As-treated population by Kaplan-Meier analysis and Cox-proportional hazard ratio analysis.

**Supplementary material 8.** Clinical outcomes of the As-treated population by Kaplan-Meier analysis and Cox-proportional hazard ratio analysis according to 3-month or 12-month DAPT strategies.

**Supplementary material 9.** Three-month landmark analyses.

**Supplementary material 10.** Time-to-event curves for NACE (A), TIMI major bleeding (B), TIMI minor bleeding (C), TIMI major or minor bleeding (D), and MACCE (E) in patients with as-treated group

**Supplementary material 11.** Independent predictors for NACE

**Supplementary material 12.** Clinical outcomes by Kaplan-Meier analysis and Cox-proportional hazard ratio analysis between STEMI and NSTEMI according to pre-PCI TIMI flow grade

**Supplementary material 13.** Univariate analysis for NACE according to the pre-PCI TIMI flow grades in comparing STEMI and NSTEMI

**Supplementary material 14.** Inclusion and exclusion criteria

**Supplementary material 15.** Univariate analysis for NACE according to the TIMI flow grades

**Supplementary material 16.** Univariate analysis for NACE according to the duration of DAPT

**Supplementary material 1.** Antiplatelet therapy during the study period

|  | Pre-PCI TIMI 0/1 | | Pre-PCI TIMI 2/3 | |
| --- | --- | --- | --- | --- |
|  | 3-month  DAPT | 12-month DAPT | 3-month  DAPT | 12-month DAPT |
| At 3 months |  |  |  |  |
| No. of patients | 572 | 546 | 464 | 459 |
| Aspirin + ticagrelor | 559 (97.7) | 530 (97.1) | 455 (98.1) | 451 (98.3) |
| Ticagrelor alone |  |  | 1 (0.2) | 0 |
| Aspirin + clopidogrel | 12 (2.1) | 15 (2.7) | 7 (1.5) | 8 (1.7) |
| Aspirin + prasugrel | 1 (0.2) | 1 (0.2) | 1 (0.2) | 0 |
| At 6 months |  |  |  |  |
| No. of patients | 565 | 542 | 462 | 457 |
| Aspirin + ticagrelor | 29 (5.1) | 504 (93.0) | 29 (6.3) | 425 (93.0) |
| Ticagrelor alone | 503 (89.0) | 0 | 402 (87.0) | 0 |
| Aspirin alone | 2 (0.4) | 9 (1.7) | 6 (1.3) | 9 (2.0) |
| Clopidogrel alone | 6 (1.1) | 0 | 1 (0.2) | 1 (0.2) |
| Aspirin + clopidogrel | 19 (3.4) | 28 (5.2) | 20 (4.3) | 21 (4.6) |
| Aspirin + prasugrel | 5 (0.9) | 1 (0.2) | 3 (0.6) | 1 (0.2) |
| Prasugrel alone | 1 (0.2) | 0 | 1 (0.2) | 0 |
| At 12 months |  |  |  |  |
| No. of patients | 560 | 537 | 456 | 447 |
| Aspirin + ticagrelor | 22 (3.9) | 467 (87.0) | 17 (3.7) | 394 (88.1) |
| Ticagrelor alone | 499 (89.1) | 9 (1.7) | 397 (87.1) | 9 (2.0) |
| Aspirin alone | 5 (0.9) | 17 (3.2) | 3 (0.7) | 11 (2.5) |
| Clopidogrel alone | 8 (1.4) | 6 (1.1) | 10 (2.2) | 5 (1.1) |
| Aspirin + clopidogrel | 21 (3.8) | 38 (7.1) | 25 (5.5) | 26 (5.8) |
| Aspirin + prasugrel | 4 (0.7) | 0 | 3 (0.7) | 2 (0.4) |
| Prasugrel alone | 1 (0.2) | 0 | 1 (0.2) | 0 |
| Total duration of DAPT, months | 3 (3-3) | 12 (12-12) | 3 (3-3) | 12 (12-12) |

Pre-PCI, pre-percutaneous coronary intervention; TIMI, Thrombolysis In Myocardial Infarction; 3-month DAPT, ticagrelor monotherapy after 3-month dual antiplatelet therapy; 12-month DAPT, ticagrelor-based 12-month dual antiplatelet therapy.

**Supplementary material 2.** Causes of non-adherence to the allocated treatment

|  | Pre-PCI TIMI 0/1 | | Pre-PCI TIMI 2/3 | |
| --- | --- | --- | --- | --- |
|  | 3-months  DAPT | 12-months DAPT | 3-months  DAPT | 12-months DAPT |
| At 3 months |  |  |  |  |
| No. of patients | 13 (2.3) | 16 (2.9) | 9 (1.9) | 8 (1.7) |
| Need for anticoagulation | 2 (0.3) | 0 | 0 | 2 (0.4) |
| Dyspnea | 4 (0.7) | 7 (1.3) | 2 (0.4) | 3 (0.6) |
| Gastrointestinal problem | 0 | 0 | 0 | 1 (0.2) |
| Angioedema | 0 | 0 | 1 (0.2) | 0 |
| Bleeding episode | 4 (0.7) | 1 (0.2) | 2 (0.4) | 2 (0.4) |
| Physicians’ discretion regarding  patients’ risks | 2 (0.3) | 8 (1.5) | 4 (0.9) |  |
| Others | 1 (0.2) |  |  |  |
| At 12 months |  |  |  |  |
| No. of patients | 61 (10.9) | 70 (13.0) | 59 (12.9) | 53 (11.9) |
| Need for anticoagulation | 2 (0.4) | 2 (0.4) |  | 1 (0.2) |
| Dyspnea | 16 (2.9) | 15 (2.8) | 14 (3.1) | 8 (1.8) |
| Gastrointestinal problem |  | 1 (0.2) |  | 1 (0.2) |
| Easy bruise | 1 (0.2) | 4 (0.7) | 2 (0.4) | 1 (0.2) |
| Angioedema |  |  | 1 (0.2) |  |
| Bleeding episode | 6 (1.1) | 9 (1.7) | 6 (1.3) | 11 (2.5) |
| Physicians’ discretion regarding  patients’ risks | 35 (6.3) | 39 (7.3) | 34 (7.5) | 31 (6.9) |
| Additional PCI |  |  | 2 (0.4) |  |
| Others | 1 (0.2) |  |  |  |

Pre-PCI, pre-percutaneous coronary intervention; TIMI, Thrombolysis In Myocardial Infarction; 3-month DAPT, ticagrelor monotherapy after 3-month dual antiplatelet therapy; 12-month DAPT, ticagrelor-based 12-month dual antiplatelet therapy.

**Supplementary material 3.** Medications during the study period.

|  | Pre-PCI TIMI 0/1 | | | Pre-PCI TIMI 2/3 | | |
| --- | --- | --- | --- | --- | --- | --- |
|  | 3-month DAPT^a^ | 12-month DAPT^b^ | p | 3-month DAPT^a^ | 12-month DAPT^b^ | p |
| At discharge |  |  |  |  |  |  |
| No. of patients | 582 | 561 |  | 475 | 465 |  |
| Beta-blockers | 389 (66.8) | 432 (77.0) | <0.001 | 304 (64.0) | 334 (71.8) | 0.010 |
| ACE inhibitors | 253 (43.5) | 295 (52.6) | 0.002 | 188 (39.6) | 203 (43.7) | 0.209 |
| ARBs | 138 (23.7) | 92 (16.4) | 0.002 | 125 (26.3) | 123 (26.5) | 0.962 |
| CCBs | 66 (11.3) | 31 (5.5) | <0.001 | 59 (12.4) | 60 (12.9) | 0.845 |
| Statin | 572 (98.3) | 552 (98.4) | 0.880 | 463 (97.5) | 458 (98.5) | 0.266 |
| At 3 months |  |  |  |  |  |  |
| No. of patients | 572 | 546 |  | 464 | 459 |  |
| Beta-blockers | 368 (64.3) | 409 (74.9) | <0.001 | 302 (65.1) | 326 (71.0) | 0.053 |
| ACE inhibitors | 180 (31.5) | 217 (39.7) | 0.004 | 119 (25.6) | 132 (28.8) | 0.301 |
| ARBs | 184 (32.2) | 129 (23.6) | 0.002 | 167 (36.0) | 172 (37.5) | 0.682 |
| CCBs | 78 (13.6) | 29 (5.3) | <0.001 | 57 (12.3) | 73 (15.9) | 0.130 |
| Statin | 555 (97.0) | 523 (95.8) | 0.264 | 447 (96.3) | 442 (96.3) | 0.974 |
| At 6 months |  |  |  |  |  |  |
| No. of patients | 565 | 542 |  | 462 | 457 |  |
| Beta-blockers | 353 (62.5) | 386 (71.2) | 0.002 | 285 (61.7) | 312 (68.3) | 0.036 |
| ACE inhibitors | 148 (26.2_ | 198 (36.5) | <0.001 | 98 (21.2) | 116 (25.4) | 0.139 |
| ARBs | 189 (33.5) | 146 (26.9) | 0.019 | 170 (36.8) | 169 (37.0) | 0.954 |
| CCBs | 84 (14.9) | 27 (5.0) | <0.001 | 68 (14.7) | 78 (17.1) | 0.367 |
| Statin | 537 (95.0) | 504 (93.0) | 0.149 | 434 (93.9) | 429 (93.9) | 0.966 |
| At 12 months |  |  |  |  |  |  |
| No. of patients | 560 | 537 |  | 456 | 447 |  |
| Beta-blockers | 333 (59.5) | 351 (65.4) | 0.044 | 269 (59.0) | 299 (66.9) | 0.014 |
| ACE inhibitors | 128 (22.9) | 176 (32.8) | <0.001 | 84 (18.4) | 102 (22.8) | 0.118 |
| ARBs | 181 (32.3) | 144 (26.8) | 0.047 | 165 (36.2) | 166 (37.1) | 0.767 |
| CCBs | 91 (16.3) | 38 (7.1) | <0.001 | 60 (13.2) | 75 (16.8) | 0.136 |
| Statin | 524 (93.6) | 495 (92.2) | 0.370 | 426 (93.4) | 415 (92.8) | 0.730 |

^a^Ticagrelor monotherapy after 3 months DAPT, ^b^Ticagrelor-based 12 months DAPT.

Pre-PCI, pre-percutaneous coronary intervention; TIMI, Thrombolysis In Myocardial Infarction; 3-month DAPT, ticagrelor monotherapy after 3-month dual antiplatelet therapy; 12-month DAPT, ticagrelor-based 12-month dual antiplatelet therapy; ACEI, angiotensin converting enzyme; ARB, angiotensin receptor blocker; CCB, calcium channel blocker.

**Supplementary material 4.** Baseline characteristics according to 3-month or 12-month DAPT strategies.

|  | Ticagrelor monotherapy after 3-mo DAPT  (n = 1057) | | | | Ticagrelor-based 12-mo DAPT group  (n = 1026) | | | |
| --- | --- | --- | --- | --- | --- | --- | --- | --- |
| Variables | Total | Pre-PCI  TIMI 0/1  (n = 582) | Pre-PCI  TIMI 2/3  (n = 475) | p | Total | Pre-PCI  TIMI 0/1  (n = 561) | Pre-PCI  TIMI 2/3  (n = 465) | p |
| Age (years) | 59.8 ± 10.9 | 58.5 ± 10.8 | 61.3 ± 10.9 | <0.001 | 60.1 ± 11.0 | 58.8 ± 10.7 | 61.7 ± 11.1 | <0.001 |
| Men, n (%) | 862 (81.6) | 476 (81.8) | 386 (81.3) | 0.827 | 855 (83.3) | 481 (85.7) | 374 (80.4) | 0.023 |
| LVEF (%) | 52.1 ± 11.2 | 49.9 ± 10.9 | 54.8 ± 11.0 | <0.001 | 51.5 ± 11.5 | 48.9 ± 10.5 | 54.4 ± 11.8 | <0.001 |
| BMI (kg/m^2^) | 24.8 ± 3.2 | 24.9 ± 3.2 | 24.7 ± 3.2 | 0.322 | 24.9 ± 3.3 | 25.0 ±3.2 | 24.8 ± 3.4 | 0.415 |
| Hypertension, n (%) | 507 (48.0) | 261 (44.8) | 246 (51.8) | 0.025 | 487 (47.5) | 243 (43.3) | 244 (52.5) | 0.003 |
| Diabetes mellitus, n (%) | 266 (25.2) | 130 (22.3) | 136 (28.6) | 0.019 | 259 (25.2) | 117 (20.9) | 142 (30.5) | <0.001 |
| Dyslipidemia, n (%) | 620 (58.7) | 332 (57.0) | 288 (60.6) | 0.239 | 598 (58.3) | 320 (57.0) | 278 (59.8) | 0.375 |
| Prior MI, n (%) | 43 (4.1) | 23 (4.0) | 20 (4.2) | 0.832 | 25 (2.4) | 9 (1.6) | 16 (3.4) | 0.068 |
| Prior PCI, n (%) | 75 (7.1) | 40 (6.9) | 35 (7.4) | 0.755 | 64 (6.2) | 25 (4.5) | 39 (8.4) | 0.013 |
| Prior CABG, n (%) | 4 (0.4) | 3 (0.5) | 1 (0.2) | 0.632 | 7 (0.7) | 2 (0.4) | 5 (1.1) | 0.255 |
| Prior HF, n (%) | 15 (1.4) | 7 (1.2) | 8 (1.7) | 0.510 | 16 (1.6) | 12 (2.1) | 4 (0.9) | 0.130 |
| Prior stroke, n (%) | 35 (3.3) | 18 (3.1) | 17 (3.6) | 0.660 | 48 (4.7) | 24 (4.3) | 24 (5.2) | 0.505 |
| Current smokers, n (%) | 442 (41.8) | 254 (43.6) | 188 (39.6) | 0.189 | 453 (44.2) | 269 (48.0) | 184 (39.6) | 0.007 |
| White blood cell (x10^9^/L) | 10.1 ± 4.0 | 10.8 ± 4.3 | 9.1 ± 3.3 | <0.001 | 10.3 ± 3.6 | 10.9 ± 3.6 | 9.5 ± 3.4 | <0.001 |
| Hemoglobin (g/dL) | 14.4 ± 1.7 | 14.5 ± 1.7 | 14.2 ± 1.8 | 0.005 | 14.5 ± 1.7 | 14.7 ± 1.7 | 14.3 ± 1.8 | <0.001 |
| Platelet (x10^9^/L) | 241.5 ± 62.6 | 244.8 ± 60.0 | 237.5 ± 65.6 | 0.062 | 245.7 ± 70.6 | 245.4 ± 65.2 | 246.0 ± 76.7 | 0.899 |
| Peak CK-MB (mg/dL) | 402.4 ± 928.7 | 476.7 ± 932.7 | 311.3 ± 920.4 | 0.086 | 360.6 ± 807.4 | 475.3 ± 970.1 | 222.2 ± 518.9 | <0.001 |
| Peak troponin-I (ng/mL) | 25.8 ± 20.8 | 38.5 ± 32.0 | 16.3 ± 27.4 | 0.096 | 23.0 ± 17.3 | 27.0 ± 23.0 | 18.1 ± 43.0 | 0.379 |
| Serum creatinine (mg/L) | 0.99 ± 0.78 | 0.97 ± 0.55 | 1.02 ± 0.84 | 0.269 | 1.09 ± 1.01 | 1.06 ± 0.93 | 1.12 ± 1.11 | 0.316 |
| eGFR (mL/min/1.73m^2^) | 78.5 ± 25.7 | 78.5 ± 22.6 | 78.4 ± 29.1 | 0.965 | 75.2 ± 23.4 | 75.4 ± 22.1 | 74.9 ± 24.9 | 0.781 |
| Clinical presentation |  |  |  |  |  |  |  |  |
| NSTEMI | 528 (50.0) | 190 (32.6) | 338 (71.2) | <0.001 | 480 (46.8) | 170 (30.3) | 310 (66.7) | <0.001 |
| STEMI | 529 (50.0) | 392 (67.4) | 137 (28.8) | <0.001 | 546 (53.2) | 391 (69.7) | 155 (33.3) | <0.001 |
| Antithrombotic drug before PCI |  |  |  |  |  |  |  |  |
| Unfractionated heparin, n (%) | 740 (70.0) | 406 (69.8) | 334 (70.3) | 0.844 | 722 (70.4) | 404 (72.0) | 318 (68.4) | 0.205 |
| LMWH, n (%) | 91 (8.6) | 47 (8.1) | 44 (9.3) | 0.494 | 90 (8.8) | 48 (8.6) | 42 (9.0) | 0.825 |
| Glycoprotein IIb/IIIa inhibitors | 89 (8.4) | 73 (12.5) | 16 (3.4) | <0.001 | 87 (8.5) | 74 (13.2) | 13 (2.8) | <0.001 |
| Antiplatelet drug before PCI |  |  |  |  |  |  |  |  |
| Aspirin, n (%) | 1024 (96.9) | 561 (96.4) | 463 (97.5) | 0.314 | 987 (96.0) | 538 (95.9) | 449 (96.6) | 0.583 |
| Clopidogrel, n (%) | 298 (28.2) | 123 (21.1) | 175 (36.8) | <0.001 | 249 (24.3) | 96 (17.1) | 153 (32.9) | <0.001 |
| Ticagrelor, n (%) | 815 (77.1) | 480 (82.5) | 335 (70.5) | <0.001 | 829 (80.8) | 471 (84.0) | 358 (77.0) | 0.005 |
| Prasugrel, n (%) | 3 (0.3) | 2 (0.3) | 1 (0.2) | 0.686 | 3 (0.3) | 3 (0.5) | 0 | 0.256 |
| Other discharge medications |  |  |  |  |  |  |  |  |
| Beta-blockers, n (%) | 693 (65.6) | 389 (66.8) | 304 (64.0) | 0.334 | 766 (74.7) | 432 (77.0) | 334 (71.8) | 0.058 |
| ACE inhibitors, n (%) | 441 (41.7) | 253 (43.5) | 188 (39.6) | 0.210 | 498 (48.5) | 295 (52.6) | 203 (43.7) | 0.005 |
| ARBs, n (%) | 263 (24.9) | 138 (23.7) | 125 (26.3) | 0.330 | 215 (21.0) | 92 (16.4) | 123 (26.5) | <0.001 |
| CCBs, n (%) | 125 (11.8) | 66 (11.3) | 59 (12.4) | 0.588 | 91 (8.9) | 31 (5.5) | 60 (12.9) | <0.001 |
| Statin, n (%) | 1035 (97.9) | 572 (98.3) | 463 (97.5) | 0.360 | 1010 (98.4) | 552 (98.4) | 458 (98.5) | 0.899 |
| Angiographic & procedural characteristics |  |  |  |  |  |  |  |  |
| Infarct-related artery |  |  |  |  |  |  |  |  |
| LM, n (%) | 24 (2.3) | 6 (1.0) | 18 (3.8) | 0.003 | 18 (1.8) | 3 (0.5) | 15 (3.2) | 0.001 |
| LAD, n (%) | 520 (49.2) | 273 (46.9) | 247 (52.0) | 0.099 | 498 (48.5) | 262 (46.7) | 236 (50.8) | 0.196 |
| LCx, n (%) | 181 (17.1) | 94 (16.2) | 87 (18.3) | 0.353 | 177 (17.3) | 110 (19.6) | 67 (14.4) | 0.031 |
| RCA, n (%) | 332 (31.4) | 209 (35.9) | 123 (25.9) | <0.001 | 333 (32.5) | 186 (32.2) | 147 (31.6) | 0.639 |
| Primary PCI | 478 (45.2) | 333 (57.2) | 145 (30.5) | <0.001 | 488 (47.6) | 333 (59.4) | 155 (33.3) | <0.001 |
| Bifurcation lesion, n (%) | 140 (13.2) | 54 (9.3) | 86 (18.1) | <0.001 | 164 (16.0) | 70 (12.5) | 94 (20.2) | 0.001 |
| Extent of CAD |  |  |  |  |  |  |  |  |
| Single-vessel disease, n (%) | 474 (44.8) | 276 (47.4) | 198 (41.7) | 0.062 | 464 (45.2) | 268 (47.8) | 196 (42.2) | 0.072 |
| Two-vessel disease, n (%) | 350 (33.1) | 187 (32.1) | 163 (34.3) | 0.453 | 315 (30.7) | 166 (29.6) | 149 (32.0) | 0.396 |
| ≥ Three-vessel, n (%) | 233 (22.0) | 119 (20.4) | 114 (24.0) | 0.166 | 247 (24.1) | 127 (22.6) | 120 (25.8) | 0.237 |
| Transfermoral approach, n (%) | 546 (51.7) | 343 (58.9) | 203 (42.7) | <0.001 | 535 (52.1) | 320 (57.0) | 215 (46.2) | 0.001 |
| Treated lesions per patient | 1.23 ± 0.50 | 1.20 ± 0.46 | 1.27 ± 0.54 | 0.026 | 1.21 ± 0.47 | 1.19 ± 0.45 | 1.24 ± 0.49 | 0.051 |
| Multi-lesion intervention, n (%) | 207 (19.6) | 100 (17.2) | 107 (22.5) | 0.029 | 192 (18.7) | 91 (16.2) | 101 (21.7) | 0.025 |
| Multi-vessel intervention, n (%) | 166 (15.7) | 77 (13.2) | 89 (18.7) | 0.014 | 158 (15.4) | 71 (12.7) | 87 (18.7) | 0.007 |
| Total number of stents per patient | 1.35 ± 0.67 | 1.33 ± 0.64 | 1.38 ± 0.71 | 0.194 | 1.33 ± 0.61 | 1.33 ± 0.62 | 1.32 ± 0.61 | 0.932 |
| Stent diameter, mean (mm) | 3.17 ± 0.45 | 3.18 ± 0.43 | 3.15 ± 0.46 | 0.219 | 3.17 ± 0.43 | 3.14 ± 0.42 | 3.21 ± 0.45 | 0.014 |
| Total stent length per patient (mm) | 34.0 ± 20.1 | 34.8 ± 19.6 | 33.1 ± 20.6 | 0.174 | 34.0 ± 19.2 | 36.4 ± 19.1 | 32.3 ± 19.4 | 0.012 |
| PRECISE-DAPT score | 21.5 ± 20.4 | 21.3 ± 20.1 | 21.8 ± 20.8 | 0.671 | 21.9 ± 19.0 | 21.8 ± 19.0 | 22.0 ± 19.0 | 0.846 |
| ≥ 25, n (%) | 246 (23.3) | 124 (21.3) | 122 (25.7) | 0.094 | 230 (22.4) | 120 (21.4) | 110 (23.7) | 0.386 |

Values are mean ± SD or n (%). The p values for continuous data obtained from analysis of the unpaired t-test. The p values for categorical data obtained from chi-square test. Pre-PCI, pre-percutaneous coronary intervention; TIMI, Thrombolysis In Myocardial Infarction; DAPT, dual antiplatelet therapy; LVEF, left ventricular ejection fraction; BMI, body mass index; MI, myocardial infarction; PCI, percutaneous coronary intervention; CABG, coronary artery bypass graft; HF, heart failure; CK-MB, creatine kinase myocardial band; eGFR, estimated glomerular filtration rate; NSTEMI, non-ST-elevation MI; LMWH, low-molecular weight heparin; ACE, angiotensin converting enzyme; ARB, angiotensin receptor blocker; CCB, calcium channel blocker; LM, left main coronary artery; LAD, left anterior descending coronary artery; LCx, left circumflex coronary artery; RCA, right coronary artery; CAD, coronary artery disease; PRECISE, Predicting Bleeding Complications in Patients Undergoing Stent Implantation and Subsequent Dual Antiplatelet Therapy.

**Supplementary material 5.** Baseline characteristics for As-treated population**.**

|  | Pre-PCI TIMI flow grade 0/1  (n = 1143) | | | | Pre-PCI TIMI flow grade 2/3  (n = 940) | | | |
| --- | --- | --- | --- | --- | --- | --- | --- | --- |
| Variables | Total | Ticagrelor monotherapy after 3-mo DAPT  (n = 563) | Ticagrelor-based  12-mo DAPT group  (n = 580) | p | Total | Ticagrelor monotherapy after 3-mo DAPT  (n = 456) | Ticagrelor-based  12-mo DAPT group  (n = 484) | p |
| Age (years) | 58.7 ± 10.8 | 58.6 ± 10.9 | 58.7 ± 10.7 | 0.855 | 61.5 ± 11.0 | 61.3 ± 10.9 | 61.7 ± 11.1 | 0.572 |
| Men, n (%) | 957 (83.7) | 462 (82.1) | 495 (85.3) | 0.133 | 760 (80.9) | 372 (81.6) | 388 (80.2) | 0.582 |
| LVEF (%) | 49.4 ± 10.7 | 49.9 ± 10.7 | 48.9 ± 10.7 | 0.106 | 54.6 ± 11.4 | 54.8 ± 11.0 | 54.4 ± 11.8 | 0.664 |
| BMI (kg/m^2^) | 24.9 ± 3.2 | 24.9 ± 3.2 | 25.0 ± 3.2 | 0.957 | 24.8 ± 3.3 | 24.7 ± 3.2 | 24.8 ± 3.4 | 0.630 |
| Hypertension, n (%) | 504 (44.1) | 255 (45.3) | 249 (42.9) | 0.421 | 490 (52.1) | 235 (51.5) | 255 (52.7) | 0.724 |
| Diabetes mellitus, n (%) | 247 (21.6) | 128 (22.7) | 119 (20.5) | 0.362 | 278 (29.6) | 129 (28.3) | 149 (30.8) | 0.432 |
| Dyslipidemia, n (%) | 652 (57.0) | 326 (57.9) | 326 (56.2) | 0.562 | 566 (60.2) | 277 (60.7) | 289 (59.7) | 0.746 |
| Prior MI, n (%) | 32 (2.8) | 22 (3.9) | 10 (1.7) | 0.031 | 36 (3.8) | 18 (3.9) | 18 (3.7) | 0.855 |
| Prior PCI, n (%) | 65 (5.7) | 38 (6.7) | 27 (4.7) | 0.160 | 74 (7.9) | 32 (7.0) | 42 (8.7) | 0.397 |
| Prior CABG, n (%) | 5 (0.4) | 3 (0.5) | 2 (0.3) | 0.682 | 6 (0.6) | 1 (0.2) | 5 (1.0) | 0.219 |
| Prior HF, n (%) | 19 (1.7) | 7 (1.2) | 12 (2.1) | 0.356 | 12 (1.3) | 8 (1.8) | 4 (0.8) | 0.252 |
| Prior stroke, n (%) | 42 (3.7) | 19 (3.4) | 23 (4.0) | 0.639 | 41 (4.4) | 15 (3.3) | 26 (5.4) | 0.150 |
| Current smokers, n (%) | 523 (45.8) | 247 (43.9) | 276 (47.6) | 0.213 | 372 (39.6) | 179 (39.3) | 193 (39.9) | 0.894 |
| White blood cell, x10^9^/L | 10.9 ± 4.0 | 10.9 ± 4.4 | 10.8 ± 3.6 | 0.855 | 9.3 ± 3.4 | 9.1 ± 3.2 | 9.5 ± 3.5 | 0.066 |
| Hemoglobin, g/dL | 14.6 ± 1.7 | 14.6 ± 1.7 | 14.7 ± 1.7 | 0.223 | 14.2 ± 1.8 | 14.2 ± 1.8 | 14.3 ± 1.8 | 0.990 |
| Platelet, x10^9^/L | 245.1 ± 62.6 | 245.5 ± 59.9 | 244.8 ± 65.2 | 0.856 | 241.7 ± 71.4 | 236.7 ± 65.2 | 246.4 ± 76.5 | 0.036 |
| Peak CK-MB (mg/dL) | 476.0 ± 950.8 | 483.7 ± 943.1 | 468.6 ± 959.0 | 0.788 | 267.2 ± 718.9 | 302.3 ± 939.9 | 234.2 ± 578.0 | 0.472 |
| Peak troponin-I (ng/mL) | 32.8 ± 27.9 | 38.8 ± 32.5 | 27.0 ± 22.7 | 0.479 | 17.2 ± 36.0 | 16.7 ± 27.9 | 17.7 ± 42.3 | 0.688 |
| Serum creatinine (mg/L) | 1.01 ± 0.76 | 0.98 ± 0.56 | 1.05 ± 0.91 | 0.078 | 1.07 ± 0.99 | 1.02 ± 0.86 | 1.12 ± 1.09 | 0.131 |
| eGFR (mL/min/1.73m^2^) | 77.0 ± 22.4 | 78.2 ± 22.5 | 75.8 ± 22.2 | 0.076 | 76.7 ± 27.1 | 78.4 ± 29.2 | 75.1 ± 25.0 | 0.062 |
| Clinical presentation |  |  |  |  |  |  |  |  |
| NSTEMI | 360 (31.5) | 182 (32.3) | 178 (30.7) | 0.551 | 648 (68.9) | 333 (70.8) | 325 (67.1) | 0.222 |
| STEMI | 783 (68.5) | 381 (67.6) | 402 (69.3) | 0.551 | 292 (31.1) | 133 (29.2) | 159 (32.9) | 0.222 |
| Antithrombotic drug before PCI |  |  |  |  |  |  |  |  |
| Unfractionated heparin, n (%) | 810 (70.9) | 385 (68.4) | 425 (73.3) | 0.069 | 652 (69.4) | 319 (70.0) | 333 (68.8) | 0.701 |
| LMWH, n (%) | 95 (8.3) | 46 (8.2) | 49 (8.4) | 0.915 | 86 (9.1) | 42 (9.2) | 44 (9.1) | 0.949 |
| Glycoprotein IIb/IIIa inhibitors | 147 (12.9) | 71 (12.6) | 76 (13.1) | 0.860 | 29 (3.1) | 15 (3.3) | 14 (2.9) | 0.851 |
| Antiplatelet drug before PCI |  |  |  |  |  |  |  |  |
| Aspirin, n (%) | 1099 (96.2) | 542 (96.3) | 557 (96.0) | 0.836 | 912 (97.0) | 445 (97.6) | 467 (96.5) | 0.321 |
| Clopidogrel, n (%) | 219 (19.2) | 120 (21.3) | 99 (17.1) | 0.072 | 328 (34.9) | 169 (37.1) | 159 (32.9) | 0.176 |
| Ticagrelor, n (%) | 951 (83.2) | 462 (82.1) | 489 (84.3) | 0.309 | 693 (73.7) | 319 (70.0) | 374 (77.3) | 0.011 |
| Prasugrel, n (%) | 5 (0.4) | 2 (0.4) | 3 (0.5) | 0.678 | 1 (0.1) | 1 (0.2) | 0 | 0.485 |
| Other discharge medications |  |  |  |  |  |  |  |  |
| Beta-blockers, n (%) | 821 (71.8) | 376 (66.8) | 445 (76.7) | <0.001 | 638 (67.9) | 296 (64.9) | 342 (70.7) | 0.059 |
| ACE inhibitors, n (%) | 603 (46.0) | 245 (43.5) | 303 (52.2) | 0.004 | 391 (41.6) | 183 (40.1) | 208 (43.0) | 0.377 |
| ARBs, n (%) | 230 (20.1) | 138 (24.5) | 92 (15.9) | <0.001 | 248 (26.4) | 118 (25.9) | 130 (26.9) | 0.767 |
| CCBs, n (%) | 97 (8.5) | 65 (11.5) | 32 (5.5) | <0.001 | 119 (12.7) | 57 (12.5) | 62 (12.8) | 0.922 |
| Statin, n (%) | 1124 (98.3) | 555 (98.6) | 569 (98.1) | 0.530 | 921 (98.0) | 447 (98.0) | 474 (97.9) | 0.920 |
| Angiographic & procedural characteristics | | | | | | | | |
| Infarct-related artery |  |  |  |  |  |  |  |  |
| LM, n (%) | 9 (0.8) | 5 (0.9) | 4 (0.7) | 0.750 | 33 (3.5) | 17 (3.7) | 16 (3.3) | 0.727 |
| LAD, n (%) | 535 (46.8) | 262 (46.5) | 273 (47.1) | 0.857 | 483 (51.4) | 236 (51.8) | 247 (51.0) | 0.825 |
| LCx, n (%) | 204 (17.8) | 92 (16.3) | 112 (19.3) | 0.216 | 154 (16.4) | 86 (18.9) | 68 (14.0) | 0.052 |
| RCA, n (%) | 395 (34.6) | 204 (36.2) | 191 (32.9) | 0.263 | 270 (28.7) | 117 (25.7) | 153 (31.6) | 0.051 |
| Primary PCI, n (%) | 666 (58.3) | 325 (57.7) | 341 (58.8) | 0.715 | 300 (31.9) | 137 (30.0) | 163 (33.7) | 0.235 |
| Bifurcation lesion, n (%) | 124 (10.8) | 50 (8.9) | 74 (12.8) | 0.037 | 180 (19.1) | 82 (18.0) | 98 (20.2) | 0.407 |
| Extent of CAD |  |  |  |  |  |  |  |  |
| Single-vessel disease, n (%) | 544 (47.6) | 265 (47.1) | 279 (48.0) | 0.767 | 394 (41.9) | 191 (41.9) | 203 (41.9) | 0.986 |
| Two-vessel disease, n (%) | 353 (30.9) | 183 (32.5) | 170 (29.3) | 0.250 | 312 (33.2) | 156 (34.2) | 156 (32.2) | 0.520 |
| ≥ Three-vessel, n (%) | 246 (21.5) | 115 (20.4) | 131 (22.6) | 0.388 | 234 (24.9) | 109 (23.9) | 125 (25.8) | 0.498 |
| Transfermoral approach, n (%) | 663 (58.0) | 334 (59.3) | 329 (56.7) | 0.373 | 418 (44.5) | 195 (42.8) | 223 (46.1) | 0.325 |
| Treated lesions per patient | 1.19 ± 0.45 | 1.20 ± 0.46 | 1.18 ± 0.45 | 0.637 | 1.25 ± 0.51 | 1.26 ± 0.54 | 1.25 ± 0.49 | 0.698 |
| Multi-lesion intervention, n (%) | 191 (16.7) | 97 (17.2) | 94 (16.2) | 0.643 | 208 (22.1) | 100 (21.9) | 108 (22.3) | 0.937 |
| Multi-vessel intervention, n (%) | 148 (12.9) | 74 (13.1) | 74 (12.8) | 0.861 | 176 (18.7) | 84 (18.4) | 92 (19.0) | 0.867 |
| Total number of stents per patient | 1.33 ± 0.63 | 1.33 ± 0.64 | 1.32 ± 0.61 | 0.867 | 1.35 ± 0.66 | 1.38 ± 0.72 | 1.33 ± 0.61 | 0.329 |
| Stent diameter, mean (mm) | 3.16 ± 0.43 | 3.18 ± 0.43 | 3.15 ± 0.42 | 0.132 | 3.18 ± 0.45 | 3.15 ± 0.46 | 3.21 ± 0.44 | 0.048 |
| Total stent length per patient (mm) | 35.1 ± 19.4 | 35.0 ± 19.8 | 35.1 ± 18.9 | 0.483 | 32.7 ± 20.0 | 32.9 ± 20.6 | 32.5 ± 19.5 | 0.731 |
| PRECISE-DAPT score | 21.6 ± 19.5 | 21.7 ± 20.3 | 21.4 ± 18.8 | 0.831 | 21.9 ± 19.9 | 22.1 ± 21.3 | 21.8 ± 18.6 | 0.815 |
| ≥ 25, n (%) | 244 (21.3) | 124 (22.0) | 120 (20.7) | 0.582 | 232 (24.7) | 120 (26.3) | 112 (23.1) | 0.259 |

Values are mean ± SD or n (%). The p values for continuous data obtained from analysis of the unpaired t-test. The p values for categorical data obtained from chi-square test. Pre-PCI, pre-percutaneous coronary intervention; TIMI, Thrombolysis In Myocardial Infarction; DAPT, dual antiplatelet therapy; LVEF, left ventricular ejection fraction; BMI, body mass index; MI, myocardial infarction; PCI, percutaneous coronary intervention; CABG, coronary artery bypass graft; HF, heart failure; CK-MB, creatine kinase myocardial band; eGFR, estimated glomerular filtration rate; NSTEMI, non-ST-elevation MI; LMWH, low-molecular weight heparin; ACE, angiotensin converting enzyme; ARB, angiotensin receptor blocker; CCB, calcium channel blocker; LM, left main coronary artery; LAD, left anterior descending coronary artery; LCx, left circumflex coronary artery; RCA, right coronary artery; CAD, coronary artery disease; PRECISE, Predicting Bleeding Complications in Patients Undergoing Stent Implantation and Subsequent Dual Antiplatelet Therapy.

**Supplementary material 6.** Baseline characteristics for As-treated population according to 3-month or 12-month DAPT strategies.

|  | Ticagrelor monotherapy after 3-mo DAPT (n = 1019) | | | | Ticagrelor-based 12-mo DAPT group (n = 1064) | | | |
| --- | --- | --- | --- | --- | --- | --- | --- | --- |
| Variables | Total | Pre-PCI  TIMI 0/1  (n = 563) | Pre-PCI  TIMI 2/3  (n = 456) | p | Total | Pre-PCI  TIMI 0/1  (n = 580) | Pre-PCI  TIMI 2/3  (n = 484) | p |
| Age (years) | 59.8 ± 10.9 | 58.6 ± 10.9 | 61.3 ± 10.9 | <0.001 | 60.1 ± 11.0 | 58.7 ± 10.7 | 61.7 ± 11.1 | <0.001 |
| Men, n (%) | 834 (81.8) | 462 (82.1) | 372 (81.6) | 0.843 | 883 (83.0) | 495 (85.3) | 388 (80.2) | 0.027 |
| LVEF (%) | 52.1 ± 11.2 | 49.9 ± 10.7 | 54.8 ± 11.0 | <0.001 | 51.4 ± 11.6 | 48.9 ± 10.7 | 54.4 ± 11.8 | <0.001 |
| BMI (kg/m^2^) | 24.8 ± 3.2 | 24.9 ± 3.2 | 24.7 ± 3.2 | 0.254 | 24.9 ± 3.3 | 25.0 ± 3.2 | 24.8 ± 3.4 | 0.500 |
| Hypertension, n (%) | 490 (48.1) | 255 (45.3) | 235 (51.5) | 0.051 | 504 (47.4) | 249 (42.9) | 255 (52.7) | 0.002 |
| Diabetes mellitus, n (%) | 257 (25.2) | 128 (22.7) | 129 (28.3) | 0.050 | 268 (25.2) | 119 (20.5) | 149 (30.8) | <0.001 |
| Dyslipidemia, n (%) | 603 (59.2) | 326 (57.9) | 277 (60.7) | 0.370 | 615 (57.8) | 326 (56.2) | 289 (59.7) | 0.262 |
| Prior MI, n (%) | 40 (3.9) | 22 (3.9) | 18 (3.9) | 0.974 | 28 (2.6) | 10 (1.7) | 18 (3.7) | 0.054 |
| Prior PCI, n (%) | 70 (7.1) | 38 (6.7) | 32 (7.0) | 0.901 | 69 (6.5) | 27 (4.7) | 42 (8.7) | 0.009 |
| Prior CABG, n (%) | 4 (0.4) | 3 (0.5) | 1 (0.2) | 0.633 | 7 (0.7) | 2 (0.3) | 5 (1.0) | 0.256 |
| Prior HF, n (%) | 15 (1.5) | 7 (1.2) | 8 (1.8) | 0.604 | 16 (1.5) | 12 (2.1) | 4 (0.8) | 0.129 |
| Prior stroke, n (%) | 34 (3.3) | 19 (3.4) | 15 (3.3) | 0.940 | 49 (4.6) | 23 (4.0) | 26 (5.4) | 0.305 |
| Current smokers, n (%) | 426 (41.8) | 247 (43.9) | 179 (39.3) | 0.142 | 469 (44.1) | 276 (47.6) | 193 (39.9) | 0.013 |
| White blood cell, x10^9^/L | 10.1 ± 4.0 | 10.9 ± 4.4 | 9.1 ± 3.2 | <0.001 | 10.2 ± 3.6 | 10.8 ± 3.6 | 9.5 ± 3.4 | <0.001 |
| Hemoglobin, g/dL | 14.4 ± 1.7 | 14.6 ± 1.7 | 14.2 ± 1.8 | 0.006 | 14.5 ± 1.7 | 14.7 ± 1.7 | 14.3 ± 1.8 | <0.001 |
| Platelet, x10^9^/L | 241.5 ± 62.4 | 245.5 ± 59.9 | 236.7 ± 65.2 | 0.027 | 245.5 ± 70.5 | 244.8 ± 65.2 | 246.4 ± 76.5 | 0.711 |
| Peak CK-MB (mg/dL) | 476.0 ± 950.8 | 483.7 ± 943.1 | 302.3 ± 939.9 | 0.068 | 267.2 ± 718.9 | 468.6 ± 959.0 | 234.2 ± 578.0 | <0.001 |
| Peak troponin-I (ng/mL) | 32.8 ± 27.9 | 38.8 ± 32.5 | 16.7 ± 27.9 | 0.109 | 17.2 ± 36.0 | 27.0 ± 22.7 | 17.7 ± 42.3 | 0.332 |
| Serum creatinine (mg/L) | 0.99 ± 0.71 | 0.98 ± 0.56 | 1.02 ± 0.86 | 0.303 | 1.08 ± 1.00 | 1.05 ± 0.91 | 1.12 ± 1.09 | 0.295 |
| eGFR (mL/min/1.73m^2^) | 78.3 ± 25.7 | 78.2 ± 22.5 | 78.4 ± 29.2 | 0.873 | 75.5 ± 23.5 | 75.8 ± 22.2 | 75.1 ± 25.0 | 0.629 |
| Clinical presentation |  |  |  |  |  |  |  |  |
| NSTEMI | 514 (50.4) | 182 (32.3) | 333 (70.8) | <0.001 | 503 (46.8) | 178 (30.7) | 325 (67.1) | <0.001 |
| STEMI | 505 (49.6) | 381 (67.6) | 133 (29.2) | <0.001 | 561 (53.2) | 402 (69.3) | 159 (32.9) | <0.001 |
| Antithrombotic drug before PCI |  |  |  |  |  |  |  |  |
| Unfractionated heparin, n (%) | 704 (69.1) | 385 (68.4) | 319 (70.0) | 0.633 | 758 (71.2) | 425 (73.3) | 333 (68.8) | 0.118 |
| LMWH, n (%) | 88 (8.6) | 46 (8.2) | 42 (9.2) | 0.576 | 93 (8.7) | 49 (8.4) | 44 (9.1) | 0.744 |
| Glycoprotein IIb/IIIa inhibitors | 86 (8.4) | 71 (12.6) | 15 (3.3) | <0.001 | 90 (8.5) | 76 (13.1) | 14 (2.9) | <0.001 |
| Antiplatelet drug before PCI |  |  |  |  |  |  |  |  |
| Aspirin, n (%) | 987 (96.9) | 542 (96.3) | 445 (97.6) | 0.230 | 1024 (96.2) | 557 (96.0) | 467 (96.5) | 0.699 |
| Clopidogrel, n (%) | 289 (28.4) | 120 (21.3) | 169 (37.1) | <0.001 | 258 (24.2) | 99 (17.1) | 159 (32.9) | <0.001 |
| Ticagrelor, n (%) | 781 (76.5) | 462 (82.1) | 319 (70.0) | <0.001 | 863 (81.1) | 489 (84.3) | 374 (77.3) | 0.004 |
| Prasugrel, n (%) | 3 (0.3) | 2 (0.4) | 1 (0.2) | 0.690 | 3 (0.3) | 3 (0.5) | 0 | 0.255 |
| Other discharge medications |  |  |  |  |  |  |  |  |
| Beta-blockers, n (%) | 672 (65.9) | 376 (66.8) | 296 (64.9) | 0.550 | 787 (74.0) | 445 (76.7) | 342 (70.7) | 0.030 |
| ACE inhibitors, n (%) | 428 (42.0) | 245 (43.5) | 183 (40.1) | 0.279 | 511 (48.0) | 303 (52.2) | 208 (43.0) | 0.003 |
| ARBs, n (%) | 256 (25.1) | 138 (24.5) | 118 (25.9) | 0.663 | 222 (20.9) | 92 (15.9) | 130 (26.9) | <0.001 |
| CCBs, n (%) | 122 (12.0) | 65 (11.5) | 57 (12.5) | 0.698 | 94 (8.8) | 32 (5.5) | 62 (12.8) | <0.001 |
| Statin, n (%) | 1002 (98.3) | 555 (98.6) | 447 (98.0) | 0.624 | 1043 (98.0) | 569 (98.1) | 474 (97.9) | 0.843 |
| Angiographic & procedural characteristics | | | | | | | | |
| Infarct-related artery |  |  |  |  |  |  |  |  |
| LM, n (%) | 22 (2.2) | 5 (0.9) | 17 (3.7) | 0.002 | 20 (1.9) | 4 (0.7) | 16 (3.3) | 0.002 |
| LAD, n (%) | 498 (48.9) | 262 (46.5) | 236 (51.8) | 0.102 | 520 (48.9) | 273 (47.1) | 247 (51.0) | 0.218 |
| LCx, n (%) | 178 (17.5) | 92 (16.3) | 86 (18.9) | 0.320 | 180 (16.9) | 112 (19.3) | 68 (14.0) | 0.026 |
| RCA, n (%) | 321 (31.5) | 204 (36.2) | 117 (25.7) | <0.001 | 344 (32.3) | 191 (32.9) | 153 (31.6) | 0.693 |
| Primary PCI | 462 (45.3) | 325 (57.7) | 137 (30.0) | <0.001 | 504 (47.4) | 341 (58.8) | 163 (33.7) | <0.001 |
| Bifurcation lesion, n (%) | 132 (13.0) | 50 (8.9) | 82 (18.0) | <0.001 | 172 (16.2) | 74 (12.8) | 98 (20.2) | 0.001 |
| Extent of CAD |  |  |  |  |  |  |  |  |
| Single-vessel disease, n (%) | 456 (44.7) | 265 (47.1) | 191 (41.9) | 0.100 | 482 (45.3) | 279 (48.0) | 203 (41.9) | 0.048 |
| Two-vessel disease, n (%) | 339(33.3) | 183 (32.5) | 156 (34.2) | 0.593 | 326 (30.6) | 170 (29.3) | 156 (32.2) | 0.317 |
| ≥ Three-vessel, n (%) | 224 (22.0) | 115 (20.4) | 109 (23.9) | 0.196 | 256 (24.1) | 131 (22.6) | 125 (25.8) | 0.222 |
| Transfermoral approach, n (%) | 529 (51.9) | 334 (59.3) | 195 (42.8) | <0.001 | 552 (51.9) | 329 (56.7) | 223 (46.1) | 0.001 |
| Treated lesions per patient | 1.23 ± 0.50 | 1.20 ± 0.46 | 1.26 ± 0.54 | 0.045 | 1.21 ± 0.47 | 1.18 ± 0.45 | 1.25 ± 0.49 | 0.028 |
| Multi-lesion intervention, n (%) | 197 (19.3) | 97 (17.2) | 100 (21.9) | 0.066 | 202 (19.0) | 94 (16.2) | 108 (22.3) | 0.012 |
| Multi-vessel intervention, n (%) | 158 (15.5) | 74 (13.1) | 84 (18.4) | 0.024 | 166 (15.6) | 74 (12.8) | 92 (19.0) | 0.007 |
| Total number of stents per patient | 1.35 ± 0.68 | 1.33 ± 0.64 | 1.38 ± 0.72 | 0.301 | 1.33 ± 0.61 | 1.32 ± 0.61 | 1.33 ± 0.61 | 0.821 |
| Stent diameter, mean (mm) | 3.17 ± 0.45 | 3.18 ± 0.43 | 3.15 ± 0.46 | 0.302 | 3.18 ± 0.43 | 3.15 ± 0.42 | 3.21 ± 0.44 | 0.029 |
| Total stent length per patient (mm) | 34.1 ± 20.2 | 35.0 ± 19.8 | 32.9 ± 20.6 | 0.098 | 33.9 ± 19.2 | 35.1 ± 18.9 | 32.5 ± 19.5 | 0.028 |
| PRECISE-DAPT score | 21.9 ± 20.7 | 21.7 ± 20.3 | 22.1 ± 21.3 | 0.777 | 21.6 ± 18.7 | 21.4 ± 18.8 | 21.8 ± 18.6 | 0.786 |
| ≥ 25, n (%) | 244 (23.9) | 124 (22.0) | 120 (26.3) | 0.110 | 232 (21.8) | 120 (20.7) | 112 (23.1) | 0.335 |

Values are mean ± SD or n (%). The p values for continuous data obtained from analysis of the unpaired t-test. The p values for categorical data obtained from chi-square test. Pre-PCI, pre-percutaneous coronary intervention; TIMI, Thrombolysis In Myocardial Infarction; DAPT, dual antiplatelet therapy; LVEF, left ventricular ejection fraction; BMI, body mass index; MI, myocardial infarction; PCI, percutaneous coronary intervention; CABG, coronary artery bypass graft; HF, heart failure; CK-MB, creatine kinase myocardial band; eGFR, estimated glomerular filtration rate; NSTEMI, non-ST-elevation MI; LMWH, low-molecular weight heparin; ACE, angiotensin converting enzyme; ARB, angiotensin receptor blocker; CCB, calcium channel blocker; LM, left main coronary artery; LAD, left anterior descending coronary artery; LCx, left circumflex coronary artery; RCA, right coronary artery; CAD, coronary artery disease; PRECISE, Predicting Bleeding Complications in Patients Undergoing Stent Implantation and Subsequent Dual Antiplatelet Therapy.

**Supplementary material 7.** Clinical outcomes of the As-treated population by Kaplan-Meier analysis and Cox-proportional hazard ratio analysis.

| Pre-PCI TIMI flow grade 0/1 (n = 1143) | | | | | | | |
| --- | --- | --- | --- | --- | --- | --- | --- |
|  | Cumulative Events (%) | | | Unadjusted | | Adjusted^a^ | |
| Outcomes | Ticagrelor monotherapy after 3-mo DAPT  (n = 563) | Ticagrelor-based  12-mo DAPT group  (n = 580) | Log-rank | HR (95% CI) | p | HR (95% CI) | p |
| NACE | 19 (3.4) | 30 (5.2) | 0.140 | 0.651 (0.367 – 1.157) | 0.143 | 0.657 (0.369 – 1.169) | 0.153 |
| TIMI bleeding |  |  |  |  |  |  |  |
| Major | 5 (0.9) | 11 (1.9) | 0.149 | 0.468 (0.163 – 1.347) | 0.159 | 0.503 (0.174 – 1.453) | 0.204 |
| Minor | 9 (1.6) | 12 (2.1) | 0.555 | 0.771 (0.325 – 1.831) | 0.556 | 0.780 (0.328 – 1.851) | 0.573 |
| Major or minor | 14 (2.5) | 23 (4.0) | 0.163 | 0.626 (0.322 – 1.216) | 0.167 | 0.665 (0.342 – 1.294) | 0.230 |
| MACCE | 14 (2.5) | 20 (3.5) | 0.345 | 0.721 (0.364 – 1.427) | 0.347 | 0.715 (0.360 – 1.420) | 0.338 |
| All-cause death | 7 (1.3) | 8 (1.4) | 0.843 | 0.902 (0.327 – 2.488) | 0.843 | 0.964 (0.348 – 2.672) | 0.944 |
| Cardiac death | 5 (0.9) | 6 (1.0) | 0.802 | 0.859 (0.262 – 2.815) | 0.802 | 0.872 (0.265 – 2.866) | 0.822 |
| Acute MI | 2 (0.4) | 5 (0.9) | 0.271 | 0.410 (0.080 – 2.115) | 0.287 | 0.291 (0.054 – 1.581) | 0.153 |
| TVR | 2 (0.4) | 5 (0.9) | 0.269 | 0.409 (0.079 – 2.108) | 0.285 | 0.385 (0.074 – 2.014) | 0.258 |
| ST | 3 (0.5) | 1 (0.2) | 0.301 | 3.102 (0.323 – 7.423) | 0.327 | 3.279 (0.340 – 31.67) | 0.305 |
| Stroke |  |  |  |  |  |  |  |
| Ischemic | 3 (0.5) | 4 (0.7) | 0.734 | 0.772 (0.173 – 3.448) | 0.734 | 0.703 (0.154 – 3.206) | 0.649 |
| Hemorrhagic | 0 | 0 | - | - |  |  |  |
| Pre-PCI TIMI flow grade 2/3 (n = 940) | | | | | | | |
|  | Cumulative Events (%) | | | Unadjusted | | Adjusted^b^ | |
| Outcomes | Ticagrelor monotherapy after 3-mo DAPT  (n = 456) | Ticagrelor-based  12-mo DAPT group  (n = 484) | Log-rank | HR (95% CI) | p | HR (95% CI) | p |
| NACE | 20 (4.4) | 36 (7.5) | 0.054 | 0.589 (0.341 – 1.017) | 0.057 | 0.631 (0.363 – 1.098) | 0.104 |
| TIMI bleeding |  |  |  |  |  |  |  |
| Major | 10 (2.2) | 22 (4.6) | 0.051 | 0.483 (0.229 – 1.020) | 0.056 | 0.521 (0.245 – 1.109) | 0.091 |
| Minor | 5 (1.1) | 19 (4.0) | 0.006 | 0.278 (0.104 – 0.745) | 0.011 | 0.319 (0.117 – 0.866) | 0.025 |
| Major or minor | 15 (3.3) | 41 (8.6) | 0.001 | 0.385 (0.213 – 0.695) | 0.002 | 0.434 (0.239 – 0.791) | 0.006 |
| MACCE | 11 (2.4) | 18 (3.8) | 0.261 | 0.652 (0.308 – 1.381) | 0.264 | 0.718 (0.336 – 1.537) | 0.394 |
| All-cause death | 6 (1.3) | 10 (2.1) | 0.384 | 0.640 (0.233 – 1.762) | 0.388 | 0.740 (0.260 – 2.104) | 0.572 |
| Cardiac death | 2 (0.4) | 4 (0.8) | 0.459 | 0.532 (0.097 – 2.906) | 0.466 | 0.487 (0.085 – 2.785) | 0.419 |
| Acute MI | 2 (0.4) | 5 (1.1) | 0.294 | 0.426 (0.083 – 2.198) | 0.308 | 0.434 (0.084 – 2.254) | 0.321 |
| TVR | 2 (0.5) | 4 (0.8) | 0.459 | 0.532 (0.097 – 2.904) | 0.466 | 0.547 (0.099 – 3.017) | 0.489 |
| ST | 2 (0.4) | 3 (0.6) | 0.704 | 0.709 (0.118 – 4.240) | 0.706 | 0.688 (0.110 – 4.316) | 0.690 |
| Stroke |  |  |  |  |  |  |  |
| Ischemic | 2 (0.4) | 0 | 0.144 | - |  |  |  |
| Hemorrhagic | 1 (0.2) | 0 | 0.303 | - |  |  |  |

^a^Adjusted by age, prior MI, serum creatinine, eGFR, and stent diameter (Table S1).

^b^Adjusted by age, male, LVEF, hypertension, diabetes mellitus, prior PCI, serum creatinine, and eGFR (Table S1).

Pre-PCI, pre-percutaneous coronary intervention; TIMI, Thrombolysis In Myocardial Infarction; DAPT, dual antiplatelet therapy; HR, hazard ratio; CI, confidence interval; NACE, net adverse clinical events; MACCE, major adverse cardiac and cerebrovascular events; MI, myocardial infarction; TVR, target vessel revascularization; ST, stent thrombosis; eGFR, estimated glomerular filtration rate; LVEF, left ventricular ejection fraction.

**Supplementary material 8.** Clinical outcomes of the As-treated population by Kaplan-Meier analysis and Cox-proportional hazard ratio analysis according to 3-month or 12-month DAPT strategies.

| Ticagrelor monotherapy after 3-mo DAPT (n=1019) | | | | | | | |
| --- | --- | --- | --- | --- | --- | --- | --- |
|  | Cumulative Events (%) | | | Unadjusted | | Adjusted^a^ | |
| Outcomes | Pre-PCI TIMI 0/1 (n = 563) | Pre-PCI TIMI 2/3 (n = 456) | Log-rank | HR (95% CI) | p | HR (95% CI) | p |
| NACE | 19 (3.4) | 20 (4.4) | 0.411 | 0.769 (0.410 – 1.441) | 0.412 | 1.244 (0.529 – 2.834) | 0.637 |
| TIMI bleeding |  |  |  |  |  |  |  |
| Major | 5 (0.9) | 10 (2.2) | 0.088 | 0.405 (0.138 – 1.185) | 0.099 | 0.273 (0.072 – 1.028) | 0.055 |
| Minor | 9 (1.6) | 5 (1.1) | 0.495 | 1.460 (0.489 – 4.355) | 0.498 | 1.332 (0.396 – 4.473) | 0.643 |
| Major or minor | 14 (2.5) | 15 (3.3) | 0.450 | 0.756 (0.365 – 1.566) | 0.451 | 0.613 (0.265 – 1.414) | 0.251 |
| MACCE | 14 (2.5) | 11 (2.4) | 0.935 | 1.033 (0.469 – 2.276) | 0.935 | 1.224 (0.529 – 2.834) | 0.637 |
| All-cause death | 7 (1.3) | 6 (1.3) | 0.924 | 0.948 (0.319 – 2.821) | 0.924 | 1.302 (0.402 – 4.214) | 0.660 |
| Cardiac death | 5 (0.9) | 2 (0.4) | 0.387 | 2.030 (0.394 – 10.47) | 0.397 | 2.196 (0.405 – 11.92) | 0.362 |
| Acute MI | 2 (0.4) | 2 (0.4) | 0.835 | 0.812 (0.114 – 5.766) | 0.835 | 1.059 (0.143 – 7.826) | 0.955 |
| TVR | 2 (0.4) | 2 (0.5) | 0.830 | 0.807 (0.114 – 5.731) | 0.831 | 0.549 (0.047 – 6.459) | 0.633 |
| ST | 3 (0.5) | 2 (0.4) | 0.827 | 1.221 (0.204 – 7.305) | 0.827 | 0.945 (0.142 – 6.284) | 0.953 |
| Stroke |  |  |  |  |  |  |  |
| Ischemic | 3 (0.5) | 2 (0.4) | 0.830 | 1.216 (0.203 – 7.276) | 0.831 | 1.499 (0.233 – 9.645) | 0.670 |
| Hemorrhagic | 0 | 1 (0.2) | 0.268 | - | - | - | - |
| Ticagrelor-based 12-mo DAPT group (n=1064) | | | | | | | |
|  | Cumulative Events (%) | | | Unadjusted | | Adjusted^b^ | |
| Outcomes | Pre-PCI TIMI 0/1 (n = 580) | Pre-PCI TIMI 2/3 (n = 484) | Log-rank | HR (95% CI) | p | HR (95% CI) | p |
| NACE | 30 (5.2) | 36 (7.5) | 0.140 | 0.696 (0.429 – 1.130) | 0.142 | 0.830 (0.501 – 1.374) | 0.469 |
| TIMI bleeding |  |  |  |  |  |  |  |
| Major | 11 (1.9) | 22 (4.6) | 0.015 | 0.417 (0.202 – 0.860) | 0.018 | 0.497 (0.233 – 1.059) | 0.070 |
| Minor | 12 (2.1) | 19 (4.0) | 0.078 | 0.528 (0.256 – 1.008) | 0.083 | 0.602 (0.283 – 1.277) | 0.186 |
| Major or minor | 23 (4.0) | 41 (8.6) | 0.003 | 0.465 (0.279 – 0.774) | 0.003 | 0.534 (0.313 – 0.909) | 0.021 |
| MACCE | 20 (3.5) | 18 (3.8) | 0.846 | 0.939 (0.497 – 1.775) | 0.846 | 1.190 (0.612 – 2.312) | 0.608 |
| All-cause death | 8 (1.4) | 10 (2.1) | 0.400 | 0.673 (0.266 – 1.705) | 0.403 | 0.915 (0.348 – 2.411) | 0.858 |
| Cardiac death | 6 (1.0) | 4 (0.8) | 0.719 | 1.260 (0.356 – 4.466) | 0.720 | 1.652 (0.455 – 6.007) | 0.446 |
| Acute MI | 5 (0.9) | 5 (1.1) | 0.787 | 0.843 (0.244 – 2.912) | 0.787 | 1.255 (0.342 – 4.611) | 0.732 |
| TVR | 5 (0.9) | 4 (0.8) | 0.940 | 1.052 (0.282 – 3.917) | 0.940 | 1.168 (0.298 – 4.583) | 0.824 |
| ST | 1 (0.2) | 3 (0.6) | 0.239 | 0.280 (0.029 – 2.694) | 0.271 | 0.376 (0.036 – 3.935) | 0.414 |
| Stroke |  |  |  |  |  |  |  |
| Ischemic | 4 (0.7) | 0 | 0.065 | - | - | - | - |
| Hemorrhagic | 0 | 0 | - | - | - | - | - |

^a^Adjusted by age, LVEF, diabetes mellitus, white blood cell, hemoglobin, STEMI, LM, and single-vessel disease (Table S2).

^b^Adjusted by age, male, hypertension, diabetes mellitus, prior MI, prior PCI, hemoglobin, STEMI, beta-blocker, ACE inhibitor, CCB, transfemoral approach, and stent diameter (Table S2)

Pre-PCI, pre-percutaneous coronary intervention; TIMI, Thrombolysis In Myocardial Infarction; DAPT, dual antiplatelet therapy; HR, hazard ratio; CI, confidence interval; NACE, net adverse clinical events; MACCE, major adverse cardiac and cerebrovascular events; MI, myocardial infarction; TVR, target vessel revascularization; ST, stent thrombosis; LVEF, left ventricular ejection fraction; STEMI ,ST-segment elevation myocardial infarction; LM, left main coronary artery; ACE, angiotensin converting enzyme; CCB, calcium channel blocker.

**Supplementary material 9.** Three-month landmark analyses.

|  | Pre-PCI TIMI 0/1 | | | | | |
| --- | --- | --- | --- | --- | --- | --- |
| Outcomes | Cumulative Events (%) | | Unadjusted | | Adjusted^a^ | |
|  | 3-mo DAPT  (n = 582) | 12-mo DAPT  (n = 561) | HR (95% CI) | p | HR (95% CI) | p |
| NACE |  |  |  |  |  |  |
| 0 – 3 months | 15 (2.6) | 15 (2.7) | 0.866 (0.422–1.779) | 0.696 | 0.877 (0.397–1.938) | 0.745 |
| 3 – 12 months | 5 (0.9) | 14 (2.5) | 0.341 (0.123–0.947) | 0.039 | 0.358 (0.128–1.003) | 0.051 |
| TIMI major bleeding |  |  |  |  |  |  |
| 0 – 3 months | 5 (0.9) | 7 (1.3) | 0.773 (0.243–2.456) | 0.662 | 0.620 (0.169–2.270) | 0.470 |
| 3 – 12 months | 0 | 4 (0.7) | - | - | - | - |
| TIMI minor bleeding |  |  |  |  |  |  |
| 0 – 3 months | 6 (1.0) | 6 (1.1) | 0.660 (0.211–2.062) | 0.475 | 0.726 (0.220–2.397) | 0.599 |
| 3 – 12 months | 4 (0.7) | 5 (0.9) | 0.765 (0.205–2.849) | 0.690 | 0.764 (0.205–2.854) | 0.689 |
| TIMI major or minor bleeding |  |  |  |  |  |  |
| 0 – 3 months | 11 (1.9) | 13 (2.3) | 0.708 (0.315–1.589) | 0.403 | 0.645 (0.275–1.513) | 0.313 |
| 3 – 12 months | 4 (0.7) | 9 (1.7) | 0.422 (0.130–1.371) | 0.151 | 0.449 (0.138–1.461) | 0.184 |
| MACCE |  |  |  |  |  |  |
| 0 – 3 months | 10 (1.7) | 9 (1.6) | 1.190 (0.482–2.937) | 0.707 | 0.925 (0.330–2.597) | 0.883 |
| 3 – 12 months | 5 (0.9) | 10 (1.8) | 0.481 (0.164–1.406) | 0.181 | 0.468 (0.159–1.376) | 0.168 |
|  | Pre-PCI TIMI 2/3 | | | | | |
| Outcomes | Cumulative Events (%) | | Unadjusted | | Adjusted^b^ | |
|  | 3-mo DAPT  (n = 475) | 12-mo DAPT  (n = 465) | HR (95% CI) | p | HR (95% CI) | p |
| NACE |  |  |  |  |  |  |
| 0 – 3 months | 15 (3.2) | 14 (3.0) | 0.397 (0.188–0.843) | 0.016 | 0.489 (0.205–1.165) | 0.106 |
| 3 – 12 months | 8 (1.7) | 19 (4.1) | 0.413 (0.181–0.943) | 0.036 | 0.422 (0.184–0.965) | 0.041 |
| TIMI major bleeding |  |  |  |  |  |  |
| 0 – 3 months | 11 (2.3) | 10 (2.2) | 0.402 (0.166–0.976) | 0.044 | 0.594 (0.147–2.394) | 0.464 |
| 3 – 12 months | 1 (0.3) | 10 (2.1) | 0.099 (0.013–0.770) | 0.027 | 0.100 (0.012–0.796) | 0.030 |
| TIMI minor bleeding |  |  |  |  |  |  |
| 0 – 3 months | 4 (0.9) | 8 (1.7) | 0.283 (0.074–1.087) | 0.066 | 0.230 (0.050–1.025) | 0.054 |
| 3 – 12 months | 1 (0.2) | 11 (2.4) | 0.088 (0.011–0.681) | 0.020 | 0.103 (0.013–0.817) | 0.031 |
| TIMI major or minor bleeding |  |  |  |  |  |  |
| 0 – 3 months | 15 (3.2) | 18 (3.9) | 0.354 (0.170–0.736) | 0.005 | 0.355 (0.152–0.826) | 0.016 |
| 3 – 12 months | 2 (0.4) | 21 (4.6) | 0.092 (0.022–0.393) | 0.001 | 0.109 (0.025–0.467) | 0.003 |
| MACCE |  |  |  |  |  |  |
| 0 – 3 months | 5 (1.1) | 5 (1.1) | 0.507 (0.142–1.808) | 0.295 | 0.235 (0.017–3.293) | 0.282 |
| 3 – 12 months | 7 (1.5) | 12 (2.6) | 0.574 (0.226–1.457) | 0.243 | 0.697 (0.267–1.823) | 0.462 |

^a^Adjusted by age, prior MI, serum creatinine, eGFR, and stent diameter (Table S1).

^b^Adjusted by age, male, LVEF, hypertension, diabetes mellitus, prior PCI, serum creatinine, and eGFR (Table S1).

Pre-PCI, pre-percutaneous coronary intervention; TIMI, Thrombolysis In Myocardial Infarction; DAPT, dual antiplatelet therapy; HR, hazard ratio; CI, confidence interval; NACE, net adverse clinical events; MACCE, major adverse cardiac and cerebrovascular events.

**Supplementary material 10.** Time-to-event curves for NACE (a), TIMI major bleeding (b), TIMI minor bleeding (c), TIMI major or minor bleeding (d), and MACCE (e) in patients with as-treated group. aHR adjusted hazard ratio, CI confidence interval, TIMI Thrombolysis In Myocardial Infarction, NACE net adverse cardiac event, MACCE major adverse cardiac and cerebrovascular event, 3-month DAPT ticagrelor monotherapy after 3 months dual antiplatelet therapy, 12-month DAPT ticagrelor-based 12 months dual antiplatelet therapy.

**
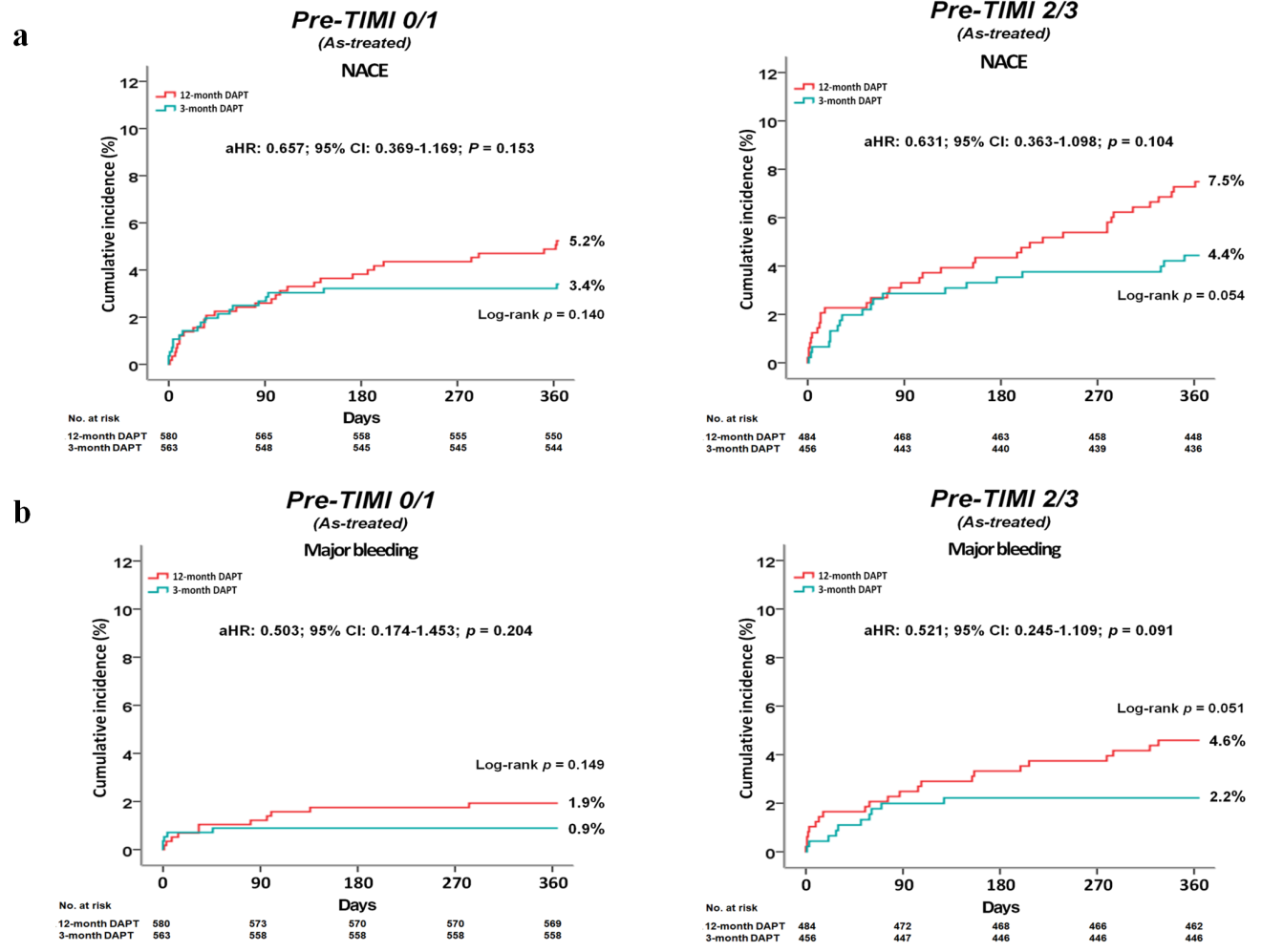
**

**
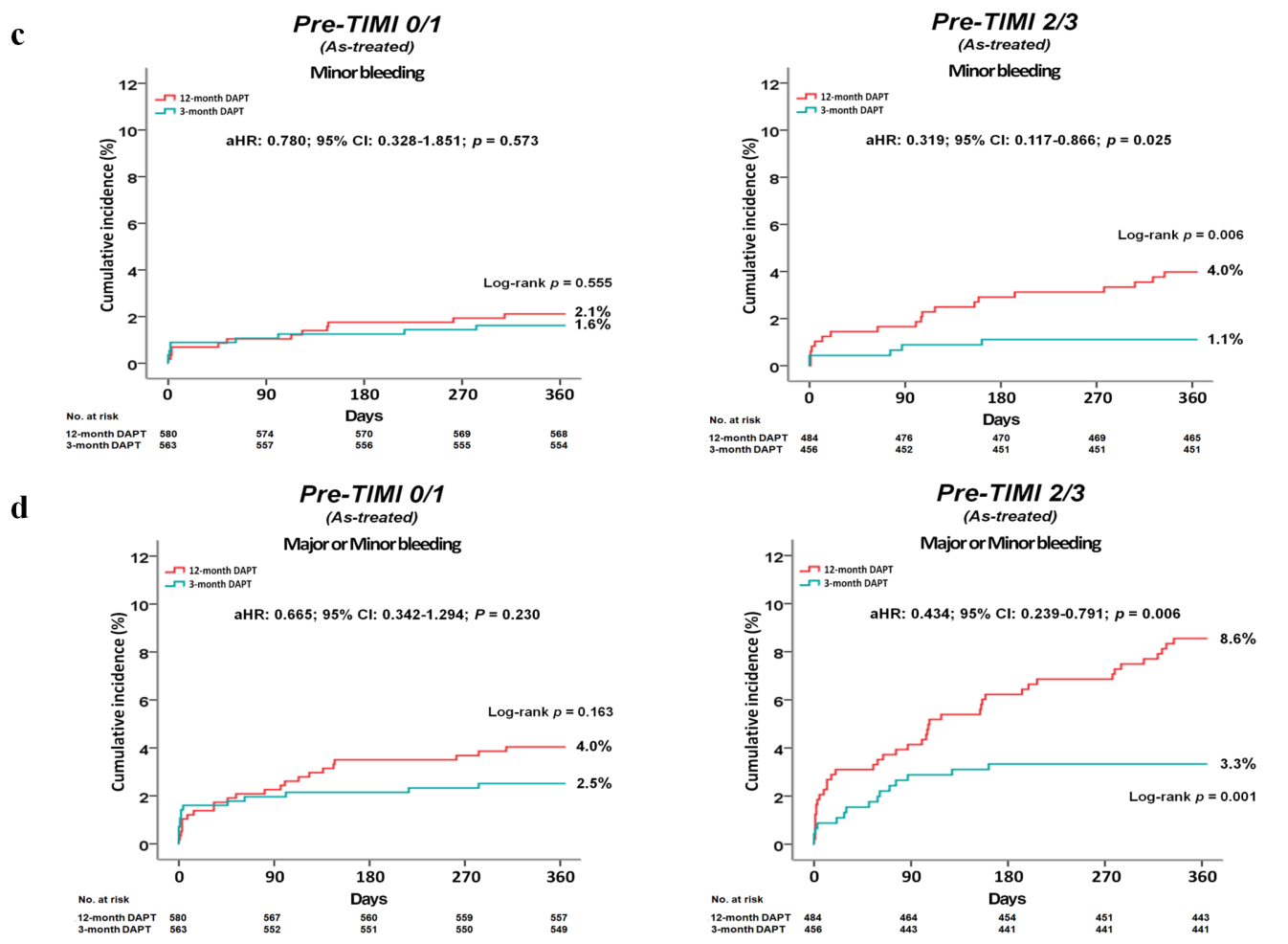
**


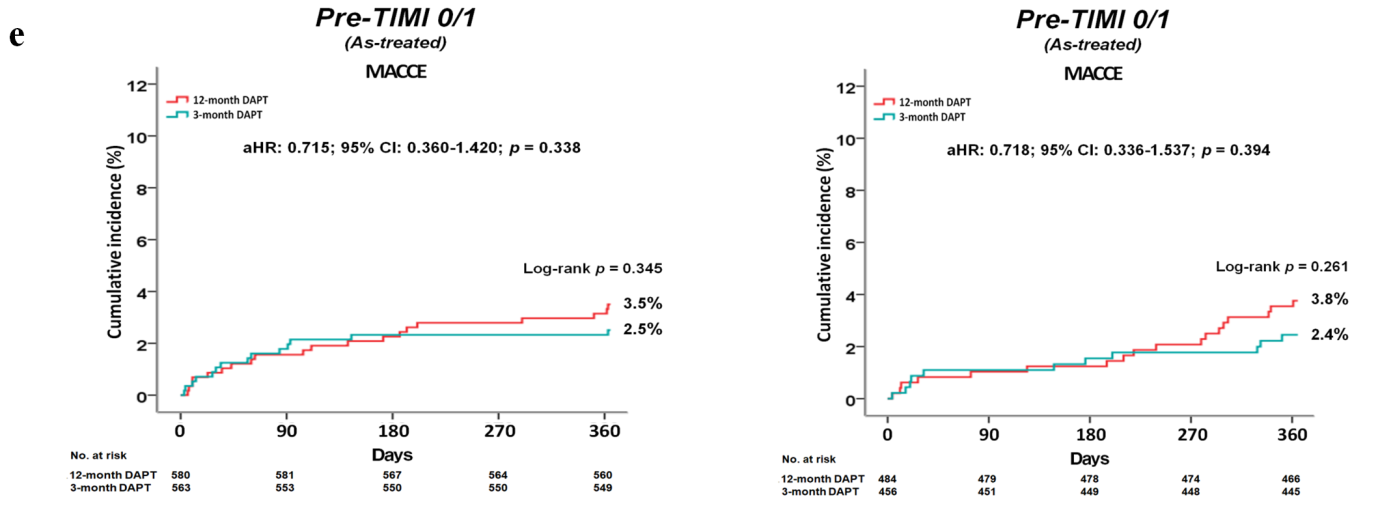


**Supplementary material 11.** Independent predictors for NACE.

|  | Univariate analysis |  | Multivariate analysis |  |
| --- | --- | --- | --- | --- |
|  | HR (95% CI) | p | HR (95% CI) | p |
| Variables | Pre-PCI TIMI 0/1 | | | |
| 3-mo vs. 12-mo DAPT | 0.663 (0.375 – 1.172) | 0.158 | 0.689 (0.389 – 1.220) | 0.201 |
| Age | 1.052 (1.023 - 1.081) | <0.001 | 1.037 (1.008 – 1.068) | 0.013 |
| Prior MI | 3.178 (1.143 - 8.836) | 0.027 | 3.172 (1.131 – 8.895) | 0.028 |
| Serum creatinine | 1.200 (1.035 - 1.391) | 0.016 | 1.035 (0.807 – 1.328) | 0.786 |
| eGFR | 0.976 (0.964 - 0.988) | <0.001 | 0.981 (0.965 – 0.998) | 0.028 |
| Stent diameter | 0.357 (0.173 - 0.736) | 0.005 | 0.361 (0.170 – 0.764) | 0.008 |
| Variables | Pre-PCI TIMI 2/3 | | | |
| 3-mo vs. 12-mo DAPT | 0.682 (0.400 – 1.161) | 0.159 | 0.741 (0.432 – 1.273) | 0.278 |
| Age | 1.040 (1.013 - 1.067) | 0.003 | 1.016 (0.986 – 1.046) | 0.299 |
| Male | 0.524 (0.296 - 0.925) | 0.026 | 0.639 (0.339 – 1.201) | 0.164 |
| LVEF | 0.975 (0.954 - 0.997) | 0.027 | 0.984 (0.963 – 1.005) | 0.137 |
| Hypertension | 2.166 (1.226 - 3.829) | 0.008 | 1.433 (0.765 – 2.683) | 0.261 |
| Diabetes mellitus | 2.849 (1.685 - 4.817) | <0.001 | 2.120 (1.196 – 3.760) | 0.010 |
| Prior PCI | 2.268 (1.111 - 4.627) | 0.024 | 1.691 (0.805 – 3.551) | 0.165 |
| Serum creatinine | 1.299 (1.165 - 1.448) | <0.001 | 1.188 (0.993 – 1.422) | 0.059 |
| eGFR | 0.981 (0.971 - 0.991) | <0.001 | 0.998 (0.985 – 1.012) | 0.804 |

NACE, net adverse clinical event; Pre-PCI, pre-percutaneous coronary intervention;TIMI, Thrombolysis In Myocardial Infarction; HR, hazard ratio; CI, confidence interval; DAPT, dual antiplatelet therapy; 3-mo, ticagrelor monotherapy after 3-mo DAPT; 12-mo, ticagrelor-based 12-mo DAPT; MI, myocardial infarction; eGFR, estimated glomerular filtration rate; LVEF, left ventricular ejection fraction.

**Supplementary material 12.** Clinical outcomes by Kaplan-Meier analysis and Cox-proportional hazard ratio analysis between STEMI and NSTEMI according to pre-PCI TIMI flow grade.

| Pre-PCI TIMI 0/1 (n = 1143) | | | | | | | |
| --- | --- | --- | --- | --- | --- | --- | --- |
|  | Cumulative Events (%) | | | Unadjusted | | Adjusted^a^ | |
| Outcomes | STEMI  (n = 783) | NSTEMI  (n = 360) | Log-rank | HR (95% CI) | p | HR (95% CI) | p |
| NACE | 29 (3.7) | 20 (5.6) | 0.160 | 0.667 (0.375 – 1.179) | 0.163 | 0.750 (0.420 – 1.340) | 0.331 |
| TIMI bleeding |  |  |  |  |  |  |  |
| Major | 9 (1.2) | 7 (2.0) | 0.293 | 0.592 (0.221 – 1.590) | 0.299 | 0.616 (0.224 – 1.695) | 0.348 |
| Minor | 16 (2.1) | 5 (1.4) | 0.438 | 1.484 (0.554 – 4.050) | 0.441 | 1.402 (0.506 – 3.888) | 0.516 |
| Major or minor | 25 (3.2) | 12 (3.4) | 0.916 | 0.964 (0.484 – 1.918) | 0.916 | 0.930 (0.461 – 1.876) | 0.839 |
| MACCE | 20 (2.6) | 14 (3.9) | 0.228 | 0.659 (0.333 – 1.304) | 0.231 | 0.761 (0.380 – 1.521) | 0.439 |
| All-cause death | 9 (1.2) | 6 (1.7) | 0.486 | 0.694 (0.247 – 1.950) | 0.488 | 0.761 (0.267 – 2.171) | 0.610 |
| Cardiac death | 8 (1.0) | 3 (0.8) | 0.757 | 1.233 (0.327 – 4.648) | 0.757 | 1.431 (0.370 – 5.542) | 0.604 |
| Acute MI | 3 (0.4) | 4 (1.1) | 0.144 | 0.344 (0.077 – 1.539) | 0.163 | 0.396 (0.087 – 1.802) | 0.231 |
| TVR | 4 (0.5) | 3 (0.9) | 0.519 | 0.614 (0.137 – 2.743) | 0.523 | 0.838 (0.181 – 3.892) | 0.822 |
| ST | 3 (0.4) | 1 (0.3) | 0.776 | 1.387 (0.144 – 13.34) | 0.777 | 1.449 (0.145 – 14.53) | 0.753 |
| Stroke |  |  |  |  |  |  |  |
| Ischemic | 5 (0.7) | 2 (0.6) | 0.864 | 1.154 (0.224 – 5.950) | 0.864 | 1.682 (0.314 – 9.023) | 0.544 |
| Hemorrhagic | 0 | 0 | - | - | - | - | - |
| Pre-PCI TIMI 2/3 (n = 940) | | | | | | | |
|  | Cumulative Events (%) | | | Unadjusted | | Adjusted^b^ | |
| Outcomes | STEMI  (n = 292) | NSTEMI  (n = 648) | Log-rank | HR (95% CI) | p | HR (95% CI) | p |
| NACE | 15 (5.2) | 41 (6.4) | 0.459 | 0.800 (0.443 – 1.445) | 0.460 | 0.706 (0.370 – 1.350) | 0.293 |
| TIMI bleeding |  |  |  |  |  |  |  |
| Major | 9 (3.1) | 23 (3.6) | 0.698 | 0.859 (0.397 – 1.856) | 0.699 | 0.717 (0.304 – 1.692) | 0.447 |
| Minor | 7 (2.4) | 17 (2.7) | 0.817 | 0.901 (0.374 – 2.174) | 0.817 | 0.780 (0.286 – 2.130) | 0.628 |
| Major or minor | 16 (5.5) | 40 (6.3) | 0.652 | 0.875 (0.490 – 1.563) | 0.652 | 0.728 (0.380 – 1.396) | 0.339 |
| MACCE | 8 (2.8) | 21(3.3) | 0.655 | 0.831 (0.368 – 1.876) | 0.656 | 0.868 (0.358 – 2.106) | 0.754 |
| All-cause death | 4 (1.4) | 12 (1.9) | 0.586 | 0.731 (0.236 – 2.267) | 0.587 | 0.909 (0.256 – 3.225) | 0.883 |
| Cardiac death | 1 (0.3) | 5 (0.8) | 0.441 | 0.440 (0.051 – 3.767) | 0.454 | 0.861 (0.082 – 9.100) | 0.901 |
| Acute MI | 2 (0.7) | 5 (0.8) | 0.873 | 0.875 (0.170 – 4.509) | 0.873 | 0.729 (0.130 – 4.095) | 0.720 |
| TVR | 3 (1.0) | 3 (0.5) | 0.326 | 2.186 (0.441 – 10.83) | 0.338 | 1.211 (0.202 – 7.251) | 0.834 |
| ST | 1 (0.3) | 4 (0.6) | 0.588 | 0.551 (0.062 – 4.929) | 0.594 | 0.975 (0.087 – 10.98) | 0.984 |
| Stroke |  |  |  |  |  |  |  |
| Ischemic | 0 | 1 (0.2) | 0.498 | - | - | - | - |
| Hemorrhagic | 0 | 1 (0.2) | 0.501 | - | - | - | - |
| Total population (n = 2083) | | | | | | | |
|  | Cumulative Events (%) | | | Unadjusted | | Adjusted^c^ | |
| Outcomes | STEMI  (n = 1075) | NSTEMI  (n = 1008) | Log-rank | HR (95% CI) | p | HR (95% CI) | p |
| NACE | 44 (4.1) | 61 (6.1) | 0.043 | 0.672 (0.456 – 0.991) | 0.045 | 0.758 (0.495 – 1.160) | 0.202 |
| TIMI bleeding |  |  |  |  |  |  |  |
| Major | 18 (1.7) | 30 (3.0) | 0.049 | 0.560 (0.312 – 1.005) | 0.052 | 0.546 (0.285 – 1.048) | 0.069 |
| Minor | 23 (2.2) | 22 (2.2) | 0.948 | 0.981 (0.547 – 1.759) | 0.948 | 0.969 (0.512 – 1.835) | 0.924 |
| Major or minor | 41 (3.9) | 52 (5.2) | 0.142 | 0.737 (0.489 – 1.109) | 0.143 | 0.745 (0.475 – 1.167) | 0.199 |
| MACCE | 28 (2.6) | 35 (3.5) | 0.252 | 0.749 (0.456 – 1.231) | 0.254 | 0.995 (0.578 – 1.714) | 0.987 |
| All-cause death | 13 (1.2) | 18 (1.8) | 0.282 | 0.678 (0.332 – 1.383) | 0.285 | 0.926 (0.414 – 2.071) | 0.851 |
| Cardiac death | 9 (0.8) | 8 (0.8) | 0.909 | 1.057 (0.408 – 2.739) | 0.909 | 1.494 (0.514 – 4.342) | 0.460 |
| Acute MI | 5 (0.5) | 9 (0.9) | 0.231 | 0.519 (0.174 – 1.549) | 0.240 | 0.589 (0.186 – 1.870) | 0.369 |
| TVR | 7 (0.7) | 6 (0.6) | 0.876 | 1.019 (0.367 – 3.246) | 0.876 | 1.131 (0.342 – 3738) | 0.840 |
| ST | 4 (0.4) | 5 (0.5) | 0.670 | 0.752 (0.202 – 2.802) | 0.672 | 0.879 (0.196 – 3.939) | 0.866 |
| Stroke |  |  |  |  |  |  |  |
| Ischemic | 5 (0.5) | 3 (0.3) | 0.538 | 1.562 (0.373 – 6.537) | 0.541 | 2.023 (0.458 – 8.941) | 0.353 |
| Hemorrhagic | 0 | 1 (0.1) | 0.303 | - | - | - | - |

^a^Adjusted by age, white blood cell, peak CK-MB, peak Troponin-I, serum creatinine, eGFR, and stent diameter (Supplementary material 13).

**Supplementary material 13.** Univariate analysis for NACE according to the pre-PCI TIMI flow grades in comparing STEMI and NSTEMI.

| Variables | Pre-PCI TIMI 0/1 | | Pre-PCI TMI 2/3 | | Pre-PCI TMI 0/1 and 2/3 | |
| --- | --- | --- | --- | --- | --- | --- |
|  | HR (95% CI) | p | HR (95% CI) | p | HR (95% CI) | p |
| Age | 1.052 (1.023 - 1.081) | <0.001 | 1.040 (1.013 - 1.067) | 0.003 | 1.047 (1.027 - 1.067) | <0.001 |
| Male | 0.535 (0.284 - 1.009) | 0.053 | 0.524 (0.296 - 0.925) | 0.026 | 0.522 (0.342 - 0.797) | 0.003 |
| LVEF | 0.976 (0.950 - 1.002) | 0.066 | 0.975 (0.954 - 0.997) | 0.027 | 0.980 (0.963 - 0.996) | 0.017 |
| Hypertension | 1.566 (0.892 - 2.750) | 0.118 | 2.166 (1.226 - 3.829) | 0.008 | 1.884 (1.268 - 2.799) | 0.002 |
| Diabetes mellitus | 1.462 (0.787 - 2.718) | 0.229 | 2.849 (1.685 - 4.817) | <0.001 | 2.183 (1.481 - 3.216) | <0.001 |
| Prior stroke | 2.394 (0.861 - 6.658) | 0.094 | 0.817 (0.199 - 3.353) | 0.780 | 1.487 (0.652 - 3.390) | 0.345 |
| Current smoker | 0.974 (0.555 - 1.710) | 0.927 | 0.841 (0.487 - 1.452) | 0.534 | 0.886 (0.599 - 1.309) | 0.543 |
| Prior PCI | 1.488 (0.535 - 4.138) | 0.446 | 2.268 (1.111 - 4.627) | 0.024 | 1.997 (1.117 - 3.570) | 0.020 |
| White blood cell | 1.052 (1.006 - 1.103) | 0.026 | 1.051 (0.977 - 1.130) | 0.185 | 1.044 (1.003 - 1.086) | 0.033 |
| Hemoglobin | 0.868 (0.744 - 1.013) | 0.072 | 0.716 (0.629 - 0.814) | <0.001 | 0.774 (0.702 - 0.853) | <0.001 |
| Platelet | 1.002 (0.998 - 1.007) | 0.301 | 1.002 (0.999 - 1.006) | 0.169 | 1.002 (1.000 - 1.005) | 0.090 |
| Peak CK-MB | 0.999 (0.995 - 1.002) | 0.445 | 0.999 (0.993 - 1.005) | 0.666 | 0.998 (0.995 - 1.001) | 0.275 |
| Peak Troponin-I | 1.000 (0.999 - 1.001) | 0.740 | 1.000 (0.999 - 1.001) | 0.971 | 1.001 (0.999 - 1.003) | 0.698 |
| Serum creatinine | 1.200 (1.035 - 1.391) | 0.016 | 1.299 (1.165 - 1.448) | <0.001 | 1.253 (1.153 - 1.362) | <0.001 |
| eGFR | 0.976 (0.964 - 0.988) | <0.001 | 0.981 (0.971 - 0.991) | <0.001 | 0.979 (0.971 - 0.987) | <0.001 |
| Unfractionated heparin | 0.701 (0.392 - 1.253) | 0.230 | 1.222 (0.677 - 2.208) | 0.506 | 0.925 (0.613 - 1.397) | 0.710 |
| Glycoprotein IIb/IIIa inhibitors | 0.435 (0.136 - 1.390) | 0.160 | 0.552 (0.076 - 3.988) | 0.556 | 0.421 (0.156 - 1.139) | 0.088 |
| Clopidogrel | 0.692 (0.311 - 1.541) | 0.368 | 1.646 (0.973 - 2.783) | 0.063 | 1.289 (0.854 - 1.946) | 0.227 |
| Ticagrelor | 1.218 (0.547 - 2.711) | 0.629 | 0.746 (0.426 - 1.308) | 0.307 | 0.853 (0.545 - 1.337) | 0.488 |
| Beta-blocker | 0.610 (0.344 - 1.085) | 0.092 | 0.666 (0.391 - 1.135) | 0.135 | 0.631 (0.427 - 0.932) | 0.021 |
| ACE inhibitor | 0.677 (0.381 - 1.203) | 0.184 | 0.704 (0.405 - 1.225) | 0.214 | 0.678 (0.455 - 1.009) | 0.055 |
| ARB | 1.451 (0.769 - 2.736) | 0.250 | 1.025 (0.568 - 1.852) | 0.934 | 1.231 (0.799 - 1.897) | 0.347 |
| CCB | 1.543 (0.657 - 3.625) | 0.320 | 1.755 (0.908 - 3.394) | 0.094 | 1.721 (1.024 - 2.893) | 0.040 |
| LAD | 1.090 (0.623 - 1.909) | 0.762 | 0.877 (0.519 - 1.481) | 0.624 | 0.986 (0.672 - 1.446) | 0.942 |
| LCx | 1.036 (0.503 - 2.135) | 0.923 | 1.100 (0.555 - 2.180) | 0.785 | 1.060 (0.645 - 1.742) | 0.819 |
| RCA | 0.834 (0.454 - 1.530) | 0.557 | 0.998 (0.559 - 1.783) | 0.996 | 0.895 (0.588 - 1.361) | 0.602 |
| Primary PCI | 0.739 (0.422 - 1.294) | 0.290 | 0.700 (0.382 - 1.282) | 0.248 | 0.675 (0.455 - 1.003) | 0.052 |
| Bifurcation lesion | 1.632 (0.765 - 3.481) | 0.205 | 0.806 (0.395 - 1.646) | 0.554 | 1.139 (0.678 - 1.914) | 0.623 |
| Transfemoral approach | 1.378 (0.765 - 2.481) | 0.286 | 2.718 (1.551 - 4.762) | <0.001 | 1.888 (1.258 - 2.832) | 0.002 |
| Multi-lesion intervention | 1.116 (0.542 - 2.301) | 0.766 | 0.861 (0.445 - 1.664) | 0.656 | 0.990 (0.608 - 1.611) | 0.967 |
| Multi-vessel intervention | 1.521 (0.738 - 3.134) | 0.256 | 0.946 (0.478 - 1.875) | 0.875 | 1.203 (0.732 - 1.977) | 0.466 |
| Treated lesions per patient | 1.058 (0.583 - 1.921) | 0.852 | 0.916 (0.536 - 1.565) | 0.748 | 0.995 (0.669 - 1.481) | 0.980 |
| Total number of stents per patient | 1.243 (0.851 - 1.814) | 0.260 | 0.926 (0.610 - 1.406) | 0.719 | 1.076 (0.812 - 1.425) | 0.611 |
| Stent diameter | 0.357 (0.173 - 0.736) | 0.005 | 0.785 (0.437 - 1.412) | 0.419 | 0.565 (0.358 - 0.892) | 0.014 |

NACE, net adverse clinical event; Pre-PCI, pre-percutaneous coronary intervention; TIMI ,Thrombolysis In Myocardial Infarction; DAPT, dual antiplatelet therapy; HR, hazard ratio; CI, confidence interval; LVEF, left ventricular ejection fraction; CK-MB, creatine kinase myocardial band; eGFR, estimated glomerular filtration rate; ACE, angiotensin converting enzyme; ARB, angiotensin receptor blocker; CCB, calcium channel blocker; LAD, left anterior descending coronary artery; LCx, left circumflex coronary artery; RCA, right coronary artery.

**Supplementary material 14.** Inclusion and exclusion criteria

| **Inclusion criteria** |
| --- |
| 1. Age ≥19 years 2. Patients who received bioresorbable polymer sirolimus-eluting stent implantation to treat acute coronary syndrome 3. Provision of informed consent |
| **Exclusion criteria** |
| 1. Age >80 years 2. Increased risk of bleeding due to    1. Any prior event of hemorrhagic stroke    2. Ischemic stroke, dementia, or impairment of central nervous system within a year    3. Traumatic brain injury or brain surgery within the past 6 months    4. Known intracranial tumor    5. Documented or suspected aortic dissection    6. Internal bleeding within the past 6 weeks    7. Active bleeding or bleeding diathesis    8. Anemia (hemoglobin ≤8 g/dL) or thrombocytopenia (platelet count < 100,000/μL)    9. Major surgery or traumatic injury resulting in any impairment of physical activity within the past 3 weeks 3. Need for oral anticoagulation therapy 4. Current or potential pregnancy 5. Life expectancy <1 year 6. Currently treated with strong cytochrome P4503A4 inhibitors 7. Moderate to severe hepatic dysfunction (Child-Pugh class B or C) 8. Increased risk of bradycardia-related symptoms |

**Supplementary material 15.** Univariate analysis for NACE according to the TIMI flow grades.

| Variables | Pre-PCI TIMI 0/1 | | Pre-PCI TIMI 2/3 | | Pre-PCI TIMI 0/1 and 2/3 | |
| --- | --- | --- | --- | --- | --- | --- |
|  | HR (95% CI) | p | HR (95% CI) | p | HR (95% CI) | p |
| Age | 1.052 (1.023 - 1.081) | <0.001 | 1.040 (1.013 - 1.067) | 0.003 | 1.047 (1.027 - 1.067) | <0.001 |
| Male | 0.535 (0.284 - 1.009) | 0.053 | 0.524 (0.296 - 0.925) | 0.026 | 0.522 (0.342 - 0.797) | 0.003 |
| LVEF | 0.976 (0.950 - 1.002) | 0.066 | 0.975 (0.954 - 0.997) | 0.027 | 0.980 (0.963 - 0.996) | 0.017 |
| Hypertension | 1.566 (0.892 - 2.750) | 0.118 | 2.166 (1.226 - 3.829) | 0.008 | 1.884 (1.268 - 2.799) | 0.002 |
| Diabetes mellitus | 1.462 (0.787 - 2.718) | 0.229 | 2.849 (1.685 - 4.817) | <0.001 | 2.183 (1.481 - 3.216) | <0.001 |
| Prior MI | 3.178 (1.143 - 8.836) | 0.027 | 1.469 (0.459 - 4.700) | 0.517 | 2.187 (1.016 - 4.709) | 0.045 |
| Prior PCI | 1.488 (0.535 - 4.138) | 0.446 | 2.268 (1.111 - 4.627) | 0.024 | 1.997 (1.117 - 3.570) | 0.020 |
| Serum creatinine | 1.200 (1.035 - 1.391) | 0.016 | 1.299 (1.165 - 1.448) | <0.001 | 1.253 (1.153 - 1.362) | <0.001 |
| eGFR | 0.976 (0.964 - 0.988) | <0.001 | 0.981 (0.971 - 0.991) | <0.001 | 0.979 (0.971 - 0.987) | <0.001 |
| Platelet | 1.002 (0.998 - 1.007) | 0.301 | 1.002 (0.999 - 1.006) | 0.169 | 1.002 (1.000 - 1.005) | 0.090 |
| STEMI | 0.667 (0.377 - 1.179) | 0.163 | 0.800 (0.443 - 1.445) | 0.460 | 0.672 (0.456 - 0.991) | 0.045 |
| Unfractionated heparin | 0.701 (0.392 - 1.253) | 0.230 | 1.222 (0.677 - 2.208) | 0.506 | 0.925 (0.613 - 1.397) | 0.710 |
| Clopidogrel | 0.692 (0.311 - 1.541) | 0.368 | 1.646 (0.973 - 2.783) | 0.063 | 1.289 (0.854 - 1.946) | 0.227 |
| Ticagrelor | 1.218 (0.547 - 2.711) | 0.629 | 0.746 (0.426 - 1.308) | 0.307 | 0.853 (0.545 - 1.337) | 0.488 |
| Beta-blocker | 0.610 (0.344 - 1.085) | 0.092 | 0.666 (0.391 - 1.135) | 0.135 | 0.631 (0.427 - 0.932) | 0.021 |
| ACE inhibitor | 0.677 (0.381 - 1.203) | 0.184 | 0.704 (0.405 - 1.225) | 0.214 | 0.678 (0.455 - 1.009) | 0.055 |
| ARB | 1.451 (0.769 - 2.736) | 0.250 | 1.025 (0.568 - 1.852) | 0.934 | 1.231 (0.799 - 1.897) | 0.347 |
| CCB | 1.543 (0.657 - 3.625) | 0.320 | 1.755 (0.908 - 3.394) | 0.094 | 1.721 (1.024 - 2.893) | 0.040 |
| LCx (treated vessel) | 1.036 (0.503 - 2.135) | 0.923 | 1.100 (0.555 - 2.180) | 0.785 | 1.060 (0.645 - 1.742) | 0.819 |
| RCA (treated vessel) | 0.834 (0.454 - 1.530) | 0.557 | 0.998 (0.559 - 1.783) | 0.996 | 0.895 (0.588 - 1.361) | 0.602 |
| Bifurcation lesion | 1.632 (0.765 - 3.481) | 0.205 | 0.806 (0.395 - 1.646) | 0.554 | 1.139 (0.678 - 1.914) | 0.623 |
| Stent diameter | 0.357 (0.173 - 0.736) | 0.005 | 0.785 (0.437 - 1.412) | 0.419 | 0.565 (0.358 - 0.892) | 0.014 |

NACE, net adverse clinical event; Pre-PCI, pre-percutaneous coronary intervention; TIMI, Thrombolysis In Myocardial Infarction; HR, hazard ratio; CI, confidence interval; LVEF, left ventricular ejection fraction; MI, myocardial infarction; eGFR, estimated glomerular filtration rate; STEMI, ST-segment elevation MI; ACE, angiotensin converting enzyme; ARB, angiotensin receptor blocker; CCB, calcium channel blocker; LCx, left circumflex coronary artery; RCA, right coronary artery.

**Supplementary material 16.** Univariate analysis for NACE according to the duration of DAPT.

| Variables | 3-month DAPT | | 12-month DAPT | | 3- and 12-month DAPT | |
| --- | --- | --- | --- | --- | --- | --- |
|  | HR (95% CI) | p | HR (95% CI) | p | HR (95% CI) | p |
| Age | 1.034 (1.005 - 1.064) | 0.023 | 1.056 (1.030 - 1.084) | <0.001 | 1.047 (1.027 - 1.067) | <0.001 |
| Male | 0.651 (0.328 - 1.291) | 0.219 | 0.439 (0.256 - 0.754) | 0.003 | 0.522 (0.342 - 0.797) | 0.003 |
| LVEF | 0.964 (0.940 - 0.989) | 0.005 | 0.992 (0.970 - 1.015) | 0.490 | 0.980 (0.963 - 0.996) | 0.017 |
| Hypertension | 1.672 (0.907 - 3.081) | 0.099 | 2.067 (1.228 - 3.477) | 0.006 | 1.884 (1.268 - 2.799) | 0.002 |
| Diabetes mellitus | 2.403 (1.316 - 4.387) | 0.004 | 2.043 (1.230 - 3.394) | 0.006 | 2.183 (1.481 - 3.216) | <0.001 |
| Prior MI | 1.807 (0.559 - 5.842) | 0.323 | 2.872 (1.043 - 7.911) | 0.041 | 2.187 (1.016 - 4.709) | 0.045 |
| Prior PCI | 1.331 (0.476 - 3.724) | 0.586 | 2.643 (1.304 - 5.358) | 0.007 | 1.997 (1.117 - 3.570) | 0.020 |
| STEMI | 0.713 (0.389 - 1.306) | 0.273 | 0.632 (0.382 - 1.047) | 0.075 | 0.672 (0.456 - 0.991) | 0.045 |
| Current smoker | 0.599 (0.313 - 1.149) | 0.123 | 1.124 (0.682 - 1.850) | 0.647 | 0.886 (0.599 - 1.309) | 0.543 |
| White blood cell | 1.057 (1.012 - 1.105) | 0.013 | 1.019 (0.953 - 1.090) | 0.588 | 1.044 (1.003 - 1.086) | 0.033 |
| Hemoglobin | 0.812 (0.697 - 0.947) | 0.008 | 0.743 (0.655 - 0.843) | <0.001 | 0.774 (0.702 - 0.853) | <0.001 |
| Platelet | 1.004 (0.999 - 1.008) | 0.086 | 1.001 (0.998 - 1.005) | 0.494 | 1.002 (1.000 - 1.005) | 0.090 |
| Peak CK-MB | 0.997 (0.992 - 1.003) | 0.370 | 0.999 (0.995 - 1.002) | 0.498 | 0.998 (0.995 - 1.001) | 0.275 |
| Peak Troponin-I | 1.000 (0.999 - 1.001) | 0.773 | 1.001 (0.999 - 1.003) | 0.823 | 1.001 (0.999 - 1.003) | 0.698 |
| Glycoprotein IIb/IIIa inhibitor | 0.523 (0.126 - 2.161) | 0.370 | 0.353 (0.087 - 1.431) | 0.145 | 0.421 (0.156 - 1.139) | 0.088 |
| Clopidogrel | 1.237 (0.654 - 2.342) | 0.513 | 1.375 (0.802 - 2.360) | 0.247 | 1.289 (0.854 - 1.946) | 0.227 |
| Ticagrelor | 0.765 (0.393 - 1.490) | 0.431 | 0.894 (0.485 - 1.647) | 0.719 | 0.853 (0.545 - 1.337) | 0.488 |
| Beta-blocker | 0.885 (0.477 - 1.643) | 0.699 | 0.452 (0.273 - 0.749) | 0.002 | 0.631 (0.427 - 0.932) | 0.021 |
| ACE inhibitor | 0.820 (0.442 - 1.522) | 0.529 | 0.565 (0.336 - 0.951) | 0.032 | 0.678 (0.455 - 1.009) | 0.055 |
| ARB | 1.479 (0.782 - 2.799) | 0.229 | 1.098 (0.605 - 1.991) | 0.759 | 1.231 (0.799 - 1.897) | 0.347 |
| CCB | 1.492 (0.664 - 3.552) | 0.333 | 2.044 (1.039 - 4.021) | 0.038 | 1.721 (1.024 - 2.893) | 0.040 |
| LM | 3.393 (1.050 - 10.97) | 0.041 | 0.958 (0.133 - 6.913) | 0.966 | 2.013 (0.741 - 5.466) | 0.170 |
| LAD | 0.892 (0.490 - 1.623) | 0.707 | 1.064 (0.647 - 1.750) | 0.808 | 0.986 (0.672 - 1.446) | 0.942 |
| LCx | 1.111 (0.516 - 2.395) | 0.788 | 1.020 (0.532 - 1.958) | 0.952 | 1.060 (0.645 - 1.742) | 0.819 |
| RCA | 0.846 (0.434 - 1.647) | 0.622 | 0.921 (0.537 - 1.581) | 0.766 | 0.895 (0.588 - 1.361) | 0.602 |
| Primary PCI | 0.775 (0.420 - 1.428) | 0.413 | 0.606 (0.360 - 1.020) | 0.059 | 0.675 (0.455 - 1.003) | 0.052 |
| Bifurcation lesion | 2.003 (0.987 - 4.064) | 0.054 | 0.673 (0.307 - 1.478) | 0.324 | 1.139 (0.678 - 1.914) | 0.623 |
| Single vessel disease | 0.365 (0.181 - 0.743) | 0.005 | 0.063 (0.645 - 1.750) | 0.812 | 0.714 (0.481 - 1.061) | 0.095 |
| Transfemoral approach | 1.310 (0.715 - 2.401) | 0.382 | 2.498 (1.430 - 4.364) | 0.001 | 1.888 (1.258 - 2.832) | 0.002 |
| Treated lesions per patient | 1.209 (0.706 - 2.072) | 0.490 | 0.835 (0.465 - 1.497) | 0.545 | 0.995 (0.669 - 1.481) | 0.980 |
| Multi-lesion intervention | 1.422 (0.717 - 2.822) | 0.313 | 0.728 (0.359 - 1.475) | 0.378 | 0.990 (0.608 - 1.611) | 0.967 |
| Multi-vessel intervention | 1.868 (0.941 - 3.705) | 0.074 | 0.809 (0.385 - 1.700) | 0.575 | 1.203 (0.732 - 1.977) | 0.466 |
| Total stent length per patient | 1.008 (0.995 - 1.021) | 0.247 | 1.002 (0.990 - 1.014) | 0.757 | 1.004 (0.996 - 1.013) | 0.323 |
| Stent diameter | 0.921 (0.469 - 1.809) | 0.811 | 0.376 (0.200 - 0.705) | 0.002 | 0.565 (0.358 - 0.892) | 0.014 |

NACE, net adverse clinical event; Pre-PCI, pre-percutaneous coronary intervention; TIMI, Thrombolysis In Myocardial Infarction; 3-month DAPT, ticagrelor monotherapy after 3-month dual antiplatelet therapy; 12-month DAPT, ticagrelor-based 12-month dual antiplatelet therapy; HR, hazard ratio; CI, confidence interval; LVEF, left ventricular ejection fraction; MI. myocardial infarction; eGFR, estimated glomerular filtration rate; STEMI, ST-segment elevation MI; CK-MB, creatine kinase myocardial band; ACE, angiotensin converting enzyme; ARB, angiotensin receptor blocker; CCB, calcium channel blocker; LM, left main coronary artery; LAD, left anterior descending coronary artery; LCx, left circumflex coronary artery; RCA, right coronary artery.
